# Bifurcations in coupled amyloid-β aggregation-inflammation systems

**DOI:** 10.1038/s41540-024-00408-7

**Published:** 2024-07-30

**Authors:** Kalyan S. Chakrabarti, Davood Bakhtiari, Nasrollah Rezaei-Ghaleh

**Affiliations:** 1 Department of Biological Science and Chemistry, Krea University, Sri City, India; 2 Stadtapotheke Calw Pharmacy, Calw, Germany; 3 Heinrich Heine University (HHU) Düsseldorf, Faculty of Mathematics and Natural Sciences, Institute of Physical Biology, Düsseldorf, Germany; 4 Institute of Biological Information Processing, IBI-7: Structural Biochemistry, Forschungszentrum Jülich, Wilhelm-Johnen-Straße, Jülich, Germany

**Keywords:** Dynamical systems, Applied mathematics

## Abstract

A complex interplay between various processes underlies the neuropathology of Alzheimer’s disease (AD) and its progressive course. Several lines of evidence point to the coupling between Aβ aggregation and neuroinflammation and its role in maintaining brain homeostasis during the long prodromal phase of AD. Little is however known about how this protective mechanism fails and as a result, an irreversible and progressive transition to clinical AD occurs. Here, we introduce a minimal model of a coupled system of Aβ aggregation and inflammation, numerically simulate its dynamical behavior, and analyze its bifurcation properties. The introduced model represents the following events: generation of Aβ monomers, aggregation of Aβ monomers into oligomers and fibrils, induction of inflammation by Aβ aggregates, and clearance of various Aβ species. Crucially, the rates of Aβ generation and clearance are modulated by inflammation level following a Hill-type response function. Despite its relative simplicity, the model exhibits enormously rich dynamics ranging from overdamped kinetics to sustained oscillations. We then specify the region of inflammation- and coupling-related parameters space where a transition to oscillatory dynamics occurs and demonstrate how changes in Aβ aggregation parameters could shift this oscillatory region in parameter space. Our results reveal the propensity of coupled Aβ aggregation-inflammation systems to oscillatory dynamics and propose prolonged sustained oscillations and their consequent immune system exhaustion as a potential mechanism underlying the transition to a more progressive phase of amyloid pathology in AD. The implications of our results in regard to early diagnosis of AD and anti-AD drug development are discussed.

## Introduction

Alzheimer’s disease (AD) is the most common cause of dementia, characterized by a progressive and irreversible loss of memory, cognitive functions, and language skills^1^. Two neuropathological hallmarks of AD are the extracellular deposition of amyloid-β (Aβ) peptide as senile plaques and intracellular deposition of tau protein as neurofibrillary tangles (NFTs)^2^. More recently, neuroinflammation has been suggested as another pathological hallmark of AD^3^. Several lines of evidence support the amyloid cascade hypothesis, according to which Aβ aggregation is the key initial event in the pathogenesis of AD, triggering a cascade of pathological events including tau protein hyperphosphorylation and aggregation into NFTs, inflammation, reactive oxygen species generation, synaptic dysfunction, and neuronal death^4^.

Aβ peptides are 39–43 residue-long peptides produced through two consecutive proteolytic cleavages of a transmembrane protein called amyloid precursor protein (APP) by membrane-bound proteases β- and γ-secretases^5^. Aβ is degraded via Aβ-degrading proteases (AβDPs) such as neprilysin, insulin-degrading enzyme, and endothelin-converting enzyme^6^, or cleared from the brain through transport to blood via blood-brain-barrier or secretion to cerebrospinal fluid (CSF)^7^. The Aβ concentration in the brain is determined by a subtle balance between its production and degradation or clearance rates. Aβ has a concentration-dependent propensity to form oligomeric and fibrillar aggregates, which, especially in the case of oligomeric aggregates, exhibit neurotoxic properties^8^. Familial forms of AD are often caused by mutations that increase the rate of total Aβ generation or the fraction of more aggregation-prone Aβ variants such as Aβ42, or lead to a change in Aβ sequence and alter its aggregation properties^9^. The molecular mechanisms underlying the sporadic forms of AD are much less known. However, the potential role of aberrations in the clearance mechanisms of Aβ and tau protein and mechanisms associated with neuroinflammation have been proposed^10,11^.

In addition to aggregation-related proteopathy, the AD brain exhibits the hallmarks of pathological inflammation^12^. As in other systems, the dynamics of the inflammatory response to an injury involves temporally coordinated transformation of pro-inflammatory to anti-inflammatory cells and, consequently, a concerted shift from pro-inflammatory to anti-inflammatory cytokines, hence the self-control of inflammatory response^13^. Several lines of evidence indicate the existence of feedback and feedforward mechanisms between Aβ (and tau protein) aggregation and inflammation, as implied in the amyloid cascade hypothesis. For example, Aβ aggregates activate astrocytes and trigger an inflammatory response through secretion of MCP-1, attraction of monocytes from blood, differentiation of monocytes to pro- and then anti-inflammatory macrophages, and coordinated activation of pro- and anti-inflammatory microglia^14–16^. On the other hand, the activated astrocytes produce Aβ^17^, and the activated macrophages and microglia promote Aβ clearance mechanisms^18^. Clearly, the coupling between Aβ aggregation and inflammation generates mechanisms compensating for the adverse effects of Aβ aggregates and maintaining brain homeostasis during the long prodromal phase of AD. Only when these compensatory mechanisms become inefficient, irreversible, and progressive transition to pre-clinical and clinical phases of AD occur^19^. Despite its crucial importance, the nature of this transition and its potential underlying molecular factors have remained elusive.

It is widely believed that amyloid aggregation of protein follows a nucleation-dependent polymerization model (NPM), in which the slow formation of growth nuclei from protein monomers precedes rapid elongation of the formed nuclei to amyloid fibrils. The actual mechanism of protein amyloid aggregation is, however, often more complex than the classical NPM^20^. For example, the formation of nuclei of aggregation may occur through alternative routes depending on whether only monomers (primary nucleation), only fibrils (fragmentation), or both monomers and fibrils (secondary nucleation) are involved. In addition, the formation of growth-competent nuclei may occur through one or more steps. The elongation step may also occur through monomer addition and end-to-end association of fibrils. In the case of Aβ, several kinetic models have been proposed to describe its in vitro aggregation, including models by Lomakin et al.^21^, Pallitto and Murphy^22^, Morris et al.^23^, and several models by Linse, Vendruscolo, and Knowles et al.^24,25^, among others. These models had proven remarkably successful in reproducing dynamics of Aβ aggregation in closed in vitro systems, especially when they were constructed on the basis of highly reproducible experimental data^25^. Little, however, is known about how the coupling with inflammation may affect the dynamics of Aβ aggregation in the in vivo context, where the rates of Aβ aggregation, as well as generation and degradation, are modulated by the existing inflammation.

Here, we present a minimal mathematical model of a coupled open system of Aβ aggregation and inflammation and analyze its dynamics by searching its parameter space. Our results demonstrate that the model can show a range of behavior—a pattern observed in nonlinear dynamical systems. The in vitro version of the model—closed and uncoupled with the immune system—reproduces the kinetic behavior observed in biochemical experiments. The open system—with reported monomeric Aβ generation and degradation rates—reproduces the physiological concentration of monomer, oligomer, and fibril. The in vivo version of the model is both open and coupled to the immune system; that is, the kinetic parameters are modulated by inflammation induced by Aβ oligomers and fibrils. This model shows the range of behaviors encompassing the previous two scenarios, but in addition, complex oscillatory behavior depends upon kinetic and coupling-related parameters. The steady oscillations over prolonged periods might lead to immune system reprogramming, exhaustion, and eventual failure^26^, which may have detrimental consequences regarding compensatory mechanisms and contribute to a transition to a more progressive phase of amyloid pathology in AD. Besides, the different roles played by the set of kinetic and coupling-related parameters in displaying oscillations might provide new candidates as potential targets for therapeutic interventions.

## Results

**Fig. 1:**
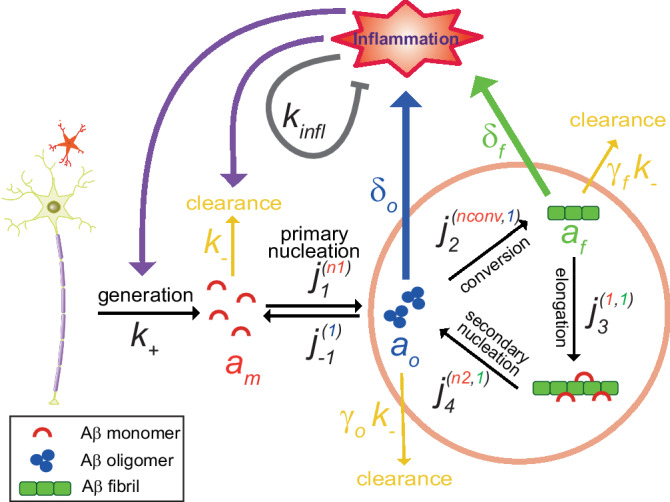
A schematic representation of the minimal model of coupled Aβ aggregation and inflammation used in this study. The following events (and related parameters) are shown: (i) generation of Aβ monomers by neurons and astrocytes (rate, ${k}_{+}$), (ii) clearance (degradation) of Aβ (rate constant for Aβ monomers, ${k}_{-}$, scaled down by factors ${\gamma }_{o}$ for oligomers and ${\gamma }_{f}$ for fibrils), (iii) primary nucleation (forward rate constant, ${j}_{1}^{n1}$, with the superscript $n1$ representing the molecular order with respect to Aβ monomers; reverse rate constant, ${j}_{-1}^{1}$, with superscript representing the molecular order of 1 with respect to Aβ oligomers), (iv) oligomer conversion (rate constant, ${j}_{2}^{{nconv},1}$, with the superscripts ${nconv}$ and 1 representing molecular orders with respect to Aβ monomers and oligomers, respectively), (v) elongation (rate constant, ${j}_{3}^{\mathrm{1,1}}$, with the superscripts 1 and 1 representing molecular orders with respect to Aβ monomers and fibrils, respectively), (vi) secondary nucleation (rate constant, ${j}_{4}^{n\mathrm{2,1}}$, with the superscripts $n2$ and 1 representing molecular orders with respect to Aβ monomers and fibrils, respectively), (vii) generation of inflammation by Aβ oligomers and fibrils (governed by the weight factors ${\delta }_{o}$ and ${\delta }_{f}$, respectively), (viii) modulation of rates of Aβ generation and clearance by inflammation, and (ix) self-inhibitory mechanisms of inflammation (represented by ${k}_{{infl}}$).

The minimal model used in this study is illustrated in Fig. 1. It contains processes of Aβ monomer generation, aggregation of Aβ monomers into oligomers and fibrils, clearance or degradation of Aβ monomers and aggregates, induction of inflammation by Aβ aggregates, and modulatory effect of inflammation on these processes. Aβ aggregation involves four steps: primary nucleation, conversion (of oligomers into elongation-competent fibrillar particles), elongation (by addition of monomers into growing ends of fibrils), and secondary nucleation (by the surface of fibrils), as described and validated in literature^25^. To represent the modulatory effect of inflammation, the rates of the above-mentioned processes were assumed to be in principle inflammation-dependent and vary from the intrinsic value in the absence of inflammation to a final value at an infinite level of inflammation according to a Hill function. The model is represented by a system of coupled ordinary differential equations (ODE). The parameters and values of the model, henceforth called standard parameter values, are listed in Table 1 (further details in “Methods”). Below, we describe the model in detail, including its constituting steps and the corresponding ODEs.

**Table 1:** The list of parameters and their standard values used in our simulations (unless the use of other values is explicitly stated)*

Parameter	Description	Value
${k}_{+}$	Rate of Aβ monomer generation:	
${k}_{+0}$	At zero inflammation level	10^−^^12^ (7.34 × 10^−^^12^)**
${k}_{+\infty }$	At infinite inflammation	0
${k}_{-}$	Rate constant of Aβ monomer clearance:	
${k}_{-0}$	At zero inflammation level	2.78 × 10^−^^5^ (2.78 × 10^−^^4^)**
${k}_{-\infty }$	At infinite inflammation	1.39 × 10^−^^4^ (1.39 × 10^−^^3^)**
${\gamma }_{o}$	Attenuation factor for Aβ clearance due to oligomer formation	0.05
${\gamma }_{f}$	Attenuation factor for Aβ clearance due to fibril formation	0.01
${j}_{1}$	Primary nucleation: association rate constant for Aβ oligomer formation	6.7 × 10^−^^8^
${j}_{-1}$	Primary nucleation: dissociation rate constant	9.7 × 10^−^^5^
$n1$	Primary nucleation: kinetic order with respect to Aβ monomers	0.8
${j}_{2}$	Oligomer conversion: forward rate constant	1.9 × 10^+9^
${j}_{-2}$	Oligomer conversion: backward rate constant	0
${nconv}$	Oligomer conversion: kinetic order with respect mmatory potentials of the oligomto Aβ monomers	2.7
${j}_{3}$	Fibril elongation: forward rate constant	6.0 × 10^+6^
${j}_{-3}$	Fibril elongation: backward rate constant	0
${j}_{4}$	Secondary nucleation: association rate constant for Aβ oligomer formation	2
${j}_{-4}$	Secondary nucleation: dissociation rate constant	0
$n2$	Secondary nucleation: kinetic order with respect to Aβ monomers	0.9
${\delta }_{o}$	Inflammation induction factor by Aβ oligomers	800,000
${\delta }_{f}$	Inflammation induction factor by Aβ fibrils	200,000
${k}_{{infl}}$	Auto-regulation constant for inflammation	0.01
${infl\_ref}$	Reference inflammation level corresponding to a response function of 0.5	0.1
${steep}$	Steepness of the response function	50

*Source: the values of Aβ aggregation-related parameters were mainly taken from model 3 in reference^25^.

**Fig. 2:**
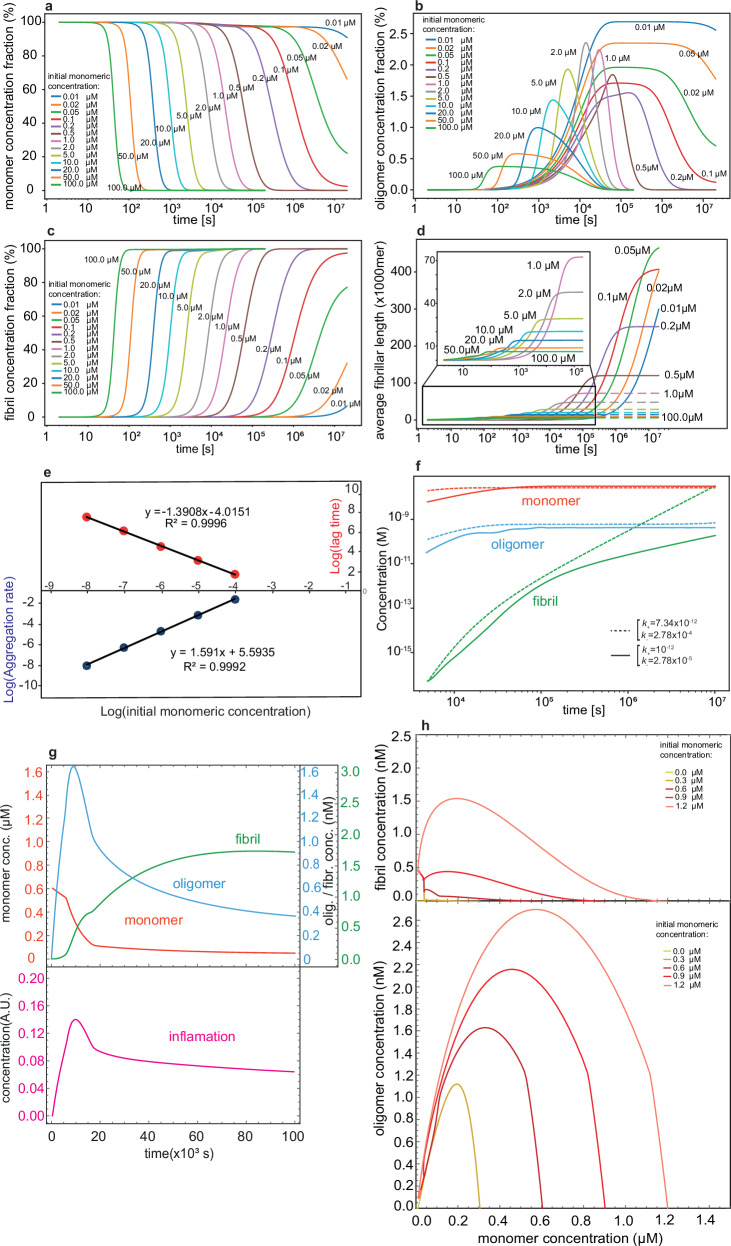
Simulated kinetics of Aβ aggregation. **a**–**d** Time-dependent changes in concentration of Aβ monomers (**a**), oligomers (**b**), and fibrils (**c**) in the closed model, i.e., with no generation and no clearance of Aβ and no inflammation or coupling with it, shown for initial Aβ monomer concentration varying from 0.01 μM to 100 μM. In **d**, the time-dependent changes in the average fibrillar length are demonstrated. **e** The log–log plots of aggregation rate and lag time vs initial Aβ monomer concentration yield apparent molecular orders (exponents) in close agreement with previous reports. **f** Time-dependent changes in concentration of Aβ monomers, oligomers, and fibrils in the open model, i.e., with generation and clearance of Aβ switched on but no inflammation or coupling with it allowed. The system approaches physiologically reasonable steady-state levels for Aβ monomers and oligomers, while gradual slight accumulation of Aβ fibrils is observed. With the two sets of generation (${k}_{+}$) and clearance (${k}_{-}$) rates used, no significant change was observed in the steady-state concentration of Aβ monomers and oligomers. **g** Time-dependent changes in concentration of Aβ monomers, oligomers, and fibrils (top panel) and inflammation level (bottom panel) in the open coupled model, i.e., an open model in which induction of inflammation and coupling between Aβ generation/clearance and inflammation are allowed. The example shown here is calculated for the initial Aβ monomer concentration of 0.6 μM. **h** Two-dimensional phase planes showing Aβ fibril (top panel) or oligomer (bottom panel) vs Aβ monomer concentration changes in the open coupled model with initial Aβ monomer concentrations of 0–1.2 μM. Despite different initial states, the trajectories converge to a fixed steady-state point.

**Fig. 3:**
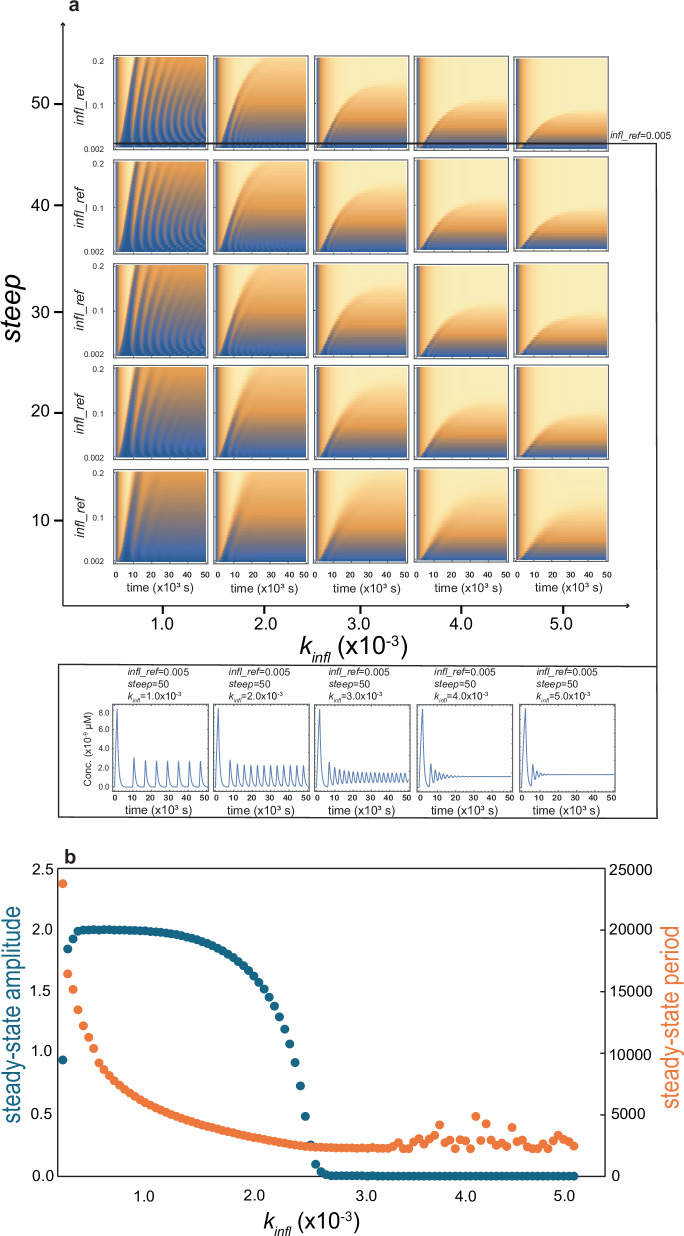
Bifurcation analysis for the coupled Aβ aggregation-inflammation with respect to inflammation- and coupling-related parameters. **a** Contour maps representing temporal changes in Aβ monomer concentration in dependence on three parameters: ${k}_{{infl}}$ (outer *x*-axis), ${steep}$ (outer *y*-axis), and ${infl\_ref}$ (inner *y*-axis). Changes in these three parameters lead to distinct kinetic behaviors. As an example, a slice of contour maps at ${steep}=50$ and ${infl\_ref}=0.005$, demonstrates how changes in the ${k}_{{infl}}$ parameter affects oscillatory changes in Aβ monomer concentration (bottom of the panel). **b** Changes in the steady-state (relative) amplitude (primary axis, blue) and period (secondary axis, orange) of oscillations caused by variation in ${k}_{{infl}}$ parameter (at ${steep}=50$ and ${infl\_ref}=0.005$). When ${k}_{{infl}}$ increases, a transition from large-amplitude slow oscillation to small-amplitude fast oscillation is observed. Oscillations with very small amplitudes represent damped oscillations, in which the calculated spacing between peaks (period) can frequently become irregular and unreliable.

**Fig. 4:**
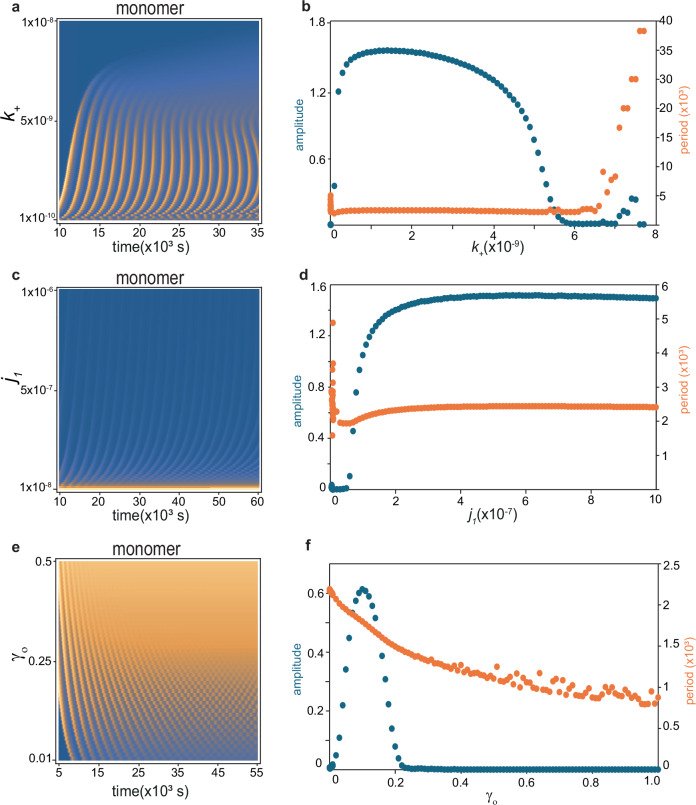
Bifurcation analysis for the coupled Aβ aggregation-inflammation system with respect to Aβ generation, aggregation, and clearance parameters. **a**–**f** Contour map representing temporal changes in Aβ monomer concentration in dependence of Aβ monomer generation rate constant ${k}_{+}$ (**a**), rate constant for Aβ oligomer formation during primary nucleation (**c**), and attenuation factor, ${\gamma }_{o}$ for clearance rate constant of Aβ oligomers relative to monomers (**e**). The corresponding changes in the steady-state (relative) amplitude (primary axis, blue) and period (secondary axis, orange) of oscillations caused by variation in these three parameters are shown in (**b**, **d**, and **f**). Pronounced changes in the oscillatory dynamics of the system are observed as a consequence of variations in these parameters (in these simulations, the values of inflammation-related parameters were: ${steep}=50$, ${infl\_ref}=0.01$ and ${k}_{{infl}}=0.003$). In panels **b**, **d**, and **f**, oscillations with very small amplitudes represent damped oscillations, in which the calculated spacing between peaks (period) can frequently become irregular and unreliable.

**To shorten the time required to reach the steady state, the parameter values in parentheses were used in calculations in the open coupled state (shown in Figs. 3 and 4). The use of these values did not lead to a significant change in the steady state calculated for the open uncoupled state (as shown in Fig. 2f).

### Equation for Aβ monomer

The Aβ monomers (${a}_{m}$) are generated and secreted at the rate ${k}_{+}$ into the extracellular space of the brain and cleared with a first-order rate constant ${k}_{-}$ with respect to Aβ monomer concentration (${a}_{m}$). The aggregation-related consumption of monomeric Aβ occurs through four distinct molecular mechanisms: (a) primary nucleation governed by the rate constant ${j}_{1}$, during which Aβ monomers form oligomers capable of progressing towards fibrillar aggregation. The kinetic order of oligomerization reaction with respect to ${a}_{m}$ is $n1$. The Aβ oligomers (${a}_{o}$) dissociate with a first-order rate constant ${j}_{-1}$, (b) the irreversible conversion of Aβ oligomers to elongation-competent fibrillar particles. This process is governed by the rate constant ${j}_{2}$ with the reaction order ${nconv}$ with respect to ${a}_{m}$ and one to ${a}_{o}$, (c) The irreversible elongation process is governed by the rate constant ${j}_{3}$, during which Aβ monomers are added to the growing ends of fibrillar particles (${a}_{{fp}}$), and (d) the irreversible secondary nucleation occurring on the surface of Aβ fibrils (${a}_{f}$), leading to the formation of new Aβ oligomers. This process is governed by the rate constant ${j}_{4}$ with the reaction order $n2$ with respect to ${a}_{m}$ and one to ${a}_{f}$. Equation 1 below describes the time-dependent changes in Aβ monomer concentration ($\dot{{a}_{m}}$) according to the above-mentioned generation, degradation, and aggregation process:

1 $$\begin{array}{l}\dot{{a}_{m}}={k}_{+}-{k}_{-}{a}_{m}-{j}_{1}{a}_{m}^{n1}+{j}_{-1}{a}_{o}\\-\frac{\,{nconv}}{\left(1+{nconv}\right)}{j}_{2}{a}_{m}^{{nconv}}{a}_{o}-{j}_{3}{a}_{m}{a}_{{fp}}-{j}_{4}{a}_{m}^{n2}{a}_{f}\end{array}$$

### Equation for Aβ oligomer

The Aβ oligomers (${a}_{o}$) are generated through reversible primary and irreversible secondary nucleation processes governed by the rate constants ${j}_{1}$, ${j}_{-1}$, and ${j}_{4}$, as described in processes (a) and (d) above. The Aβ oligomers are consumed through the irreversible aggregation-related conversion process governed by rate constant ${j}_{2}$, as in process (b) described above, and cleared following the rate constant ${k}_{-}$ scaled by an oligomer-specific factor (${\gamma }_{o}$). Equation 2 below describes the time-dependent changes in Aβ oligomer concentration ($\dot{{a}_{o}}$) according to the processes:

2 $$\dot{{a}_{o}}={j}_{1}{a}_{m}^{n1}-{j}_{-1}{a}_{o}-\frac{1}{\left(1+{nconv}\right)}{j}_{2}{a}_{m}^{{nconv}}{a}_{o}+{j}_{4}{a}_{m}^{n2}{a}_{f}-{\gamma }_{o}{k}_{-}{a}_{o}$$

### Equation for Aβ fibril and fibril particle

Time-dependent changes in concentration of Aβ fibrils are described through two variables, ${a}_{{fp}}$ and ${a}_{f}$, respectively, representing the number and mass concentration of Aβ fibrils. In our model, Aβ fibril particles, ${a}_{{fp}}$, are generated from Aβ oligomers and monomers through the irreversible conversion process, as described in process (b) above, according to:

3 $$\dot{{a}_{{fp}}}={j}_{2}{a}_{m}^{{nconv}}{a}_{o}$$

The irreversible elongation process (process c above) does not change the number concentration of fibril particles; however, it increases the mass concentration of Aβ fibrils (${a}_{f}$). In addition, we assume a clearance process for Aβ fibrils which reduces ${a}_{f}$ (but not ${a}_{{fp}}$), following the rate constant ${k}_{-}$, scaled by a fibril-specific factor (${\gamma }_{f}$).

4 $$\dot{{a}_{f}}={j}_{2}{a}_{m}^{{nconv}}{a}_{o}+{j}_{3}{a}_{m}{a}_{{fp}}-{\gamma }_{f}{k}_{-}{a}_{f}$$

### Equation for inflammation level

In our minimal model, the level of inflammation is simply represented by a single variable ${infl}$. It is assumed that inflammation is induced in a concentration-dependent manner by Aβ oligomers (${a}_{o}$) and fibril (${a}_{f}$), each weighted by a factor representing their inflammation-inducing propensity (${\delta }_{o}$ and ${\delta }_{f}$, respectively). The complex self-control of inflammation is simply modeled by a linear control mechanism involving only a proportional term with the proportionality constant ${k}_{{infl}}$.

5 $${{infl}}={\delta }_{o}{a}_{o}+{\delta }_{f}{a}_{f}-{k}_{{infl}}{infl}$$

### Coupling between inflammation and kinetic parameters

To model the effect of inflammation on various kinetic parameters, we introduce a Hill-type response function (${resp}$),

6 $${resp}\,=\,\frac{{{infl}}^{{steep}}}{{{infl}{\_}{ref}}^{{steep}}\,+\,{{infl}}^{{steep}}}$$

which varies between 0 (at ${infl}=0$) and 1 (as ${infl}\to \infty$). The parameter ${infl\_ref}$ determines a reference inflammation level at which the response function is half-maximum (i.e., ${resp}=0.5$), and the parameter ${steep}$ determines the steepness of ${resp}$ variation around this reference inflammation level. The inflammation dependence of the kinetic parameters *c* (*c*: ${k}_{+},\,{k}_{-},\,{j}_{1}{etc}.$) is in principle controlled in our model through the following relations:

7 $$c={c}_{0}+\left({c}_{{{\infty }}}-{c}_{0}\right){resp}$$

in which ${c}_{0}$ represents the intrinsic value of the kinetic parameter in the absence of inflammation and ${c}_{\infty }$, its value when ${infl}\to \infty$. While our model is capable of containing the modulatory effect of inflammation on all the interested kinetic parameters, for the sake of simplicity, we focus here on its effects on generation and degradation rate constants, ${k}_{+}$ and ${k}_{-}$. Except for a few small differences introduced because of mass conservation requirements in the closed system, the minimal model is reduced to the kinetic model introduced in the literature^25^ for in vitro aggregation of Aβ, i.e., when ${k}_{+}={k}_{-}=0$ (the system is closed) and no inflammation is present (${infl}=0$). Below, we describe the kinetic behavior of the minimal model in different systems.

### The closed uncoupled system

First, we investigated the behavior of the model when it is closed (${k}_{+}={k}_{-}=0$) and in the absence of inflammation (${infl}=0$), as shown in Fig. 2. Aβ aggregation under this condition resembles the in vitro aggregation of Aβ, which has been extensively studied through various experimental approaches^21–25^. Here, we have chosen parameter values reported in the literature^25^, where the choice of Aβ aggregation model and related parameter values was based on rigorous mass quantification of in vitro Aβ aggregation through ^3^H labeling and natural abundance mass spectrometry measurements (Table 1). The time-dependent variation in the relative amount of Aβ monomers (Fig. 2a), oligomers (Fig. 2b), and fibrils (Fig. 2c) was determined based on a range of initial monomer concentration varying from 0.01 μM up to 100 μM. The Aβ fibril formation is an irreversible process, so all the monomer is eventually converted to fibril in the course of the simulation. However, during the simulation period, Aβ oligomers were transiently formed and consumed, either dissociating back into monomers or aggregating into fibrils. The relative population and lifetime of oligomers are maximum at the lowest initial monomer concentration of 0.01 μM. At this starting concentration, the relative oligomer population reaches 2.5% from 10^5^ s to beyond 10^7^ s. On the other hand, at the highest initial monomer concentration of 100 μM, the relative population of oligomers reached only 0.5% over a much shorter period between 10^2^ s and 10^4^ s. At 1 μM, 2 μM, and 5 μM of Aβ monomer concentrations, the relative population of oligomers peaked at about 2% and fell rapidly afterward. The concentration-dependent change in the relative population and lifetime of Aβ oligomers reflect the changing relative contribution of the aggregation pathways, that is, dominated by primary nucleation at lower concentration and secondary nucleation at higher concentration. Accordingly, a transition in the calculated size of the individual fibrils was evident (Fig. 2d). The fibrils were the largest but took the longest time to reach the length of $4\times {10}^{5}$ molecules as the monomer concentration dropped to 0.05 μM. On the other hand, at concentrations above 1 μM, the fibrils did not grow beyond 50,000 monomers in length. At concentrations lower than 0.05 μM, the fibrils did not reach their full length within the simulation time of 10^7^ s.

The slopes in the Double-Logarithmic plot were then used to determine the molecular order for aggregation rate and lag time (Fig. 2e). The molecular order (exponent) was 1.59 for the aggregation rate and −1.39 for the lag time, in agreement with previous reports^24,25^. Overall, our model captures the kinetics of in vitro Aβ aggregation when the system is closed and in the absence of inflammation.

### The open uncoupled system

Next, we opened the system by switching on the generation/degradation events (${k}_{+}$ and ${k}_{-}$, respectively) and simulated the kinetic behavior of the model in the absence of inflammation or coupling with it. For ${k}_{+}$, a value of ${10}^{-12}{\rm{M.{s}}}^{-1}$ was used, which considering the total neuron number of ca. ${10}^{11}$ in human brains and a brain volume of ca. 1.5 L, corresponds roughly to 10 Aβ molecules per neuron per second, close to experimental reports^27,28^. For ${k}_{-}$, we assumed a fractional clearance rate of 10% ${h}^{-1}$, close to the values of 6–9% ${h}^{-1}$ previously reported^10,29^, which corresponds to ${k}_{-}$ of $2.78\times {10}^{-5}{\rm{M}}^{-1}{\rm{s}}^{-1}$. For the clearance of Aβ oligomers and fibrils, we used scaling factors ${\gamma }_{o}$ of 0.05 and ${\gamma }_{f}$ of 0.01, respectively, representing the expectedly higher resistance of Aβ oligomers and especially fibrils to degradation and clearance mechanisms, when compared to Aβ monomers^30^. With the used values of ${k}_{+}$ and ${k}_{-}$, a quasi-steady-state value of about 35 nM for monomeric Aβ was reached within 10^5^ s when the initial monomeric Aβ varied in the range 0–10 μM (Fig. 2f, the initial concentration for oligomeric and fibrillar Aβ concentration was zero). The steady-state level of oligomeric Aβ was 3-4 orders of magnitude smaller than that of monomeric Aβ, and only a small fraction of monomeric Aβ (about 1% when starting from 1 μM monomer) was converted to fibrils during the course of the simulation. These values are in reasonable qualitative agreement with the level of various Aβ species in young non-AD brains^31^. The steady-state concentration of Aβ lies close but below the range of 50–500 nM in which the synaptic- (but not neuro-) toxic effects of Aβ starts,^28,32^ therefore it can represent the boundary between the health state and the earliest stages of the long prodromal phase of AD. Notably, the calculated final concentration of Aβ monomers, oligomers, and especially fibrils showed relatively high sensitivity to parameters ${k}_{+}$ and ${k}_{-}$, when compared with the other model parameters (Table 1).

### The open-coupled system

Having established the intrinsic values of kinetic parameters for Aβ generation, aggregation, and degradation, the open Aβ aggregation system was subsequently coupled to the inflammation system. The oligomers and, to a smaller extent, fibrils of Aβ were allowed to induce inflammation (${\delta }_{o} > {\delta }_{f} > 0$, see Eq. 5 above), which, in turn, can modulate the kinetic parameters of the generation and degradation of Aβ through a Hill-type response function (see Eqs. 6 and 7 above). The higher propensity of small Aβ oligomers than fibrils to induce in vivo inflammation^33,34^ and the inflammation-induced increase in Aβ clearance rates^11,13,18^ are in line with previous reports. The simulated kinetic of the open coupled system using standard values of system parameters (see Table 1) showed convergence to a homeostatic state (Fig. 2g). Notably, as more clearly seen in their phase space behavior, the Aβ monomer and oligomer concentrations showed overdamping features; despite starting from different initial states and some initial spikes during the transient phase, they converged to a fixed steady-state point (Fig. 2h). When compared with the open uncoupled system, the calculated final concentration of Aβ monomers and oligomers showed much lower sensitivity to parameters ${k}_{+}$ and ${k}_{-}$ (Supplementary Table 2), reflecting the homeostatic nature of the coupled system.

Next, we studied the open-coupled system with respect to three parameters related to inflammation response, namely, ${steep}$, ${infl\_ref}$ and ${k}_{{infl}}$. Depending upon the values of these three coupling-related parameters, the system showed more complex behavior than previously observed in closed or open uncoupled systems above. While the coupled system reproduced the overdamped and damped oscillations observed in the uncoupled systems, the steady oscillations were also observed at specific values of the inflammatory parameters. Therefore, we investigated the transition from overdamped and damped oscillation to steady-oscillation (i.e., bifurcation) with respect to these coupling-related parameters as well as parameters related to Aβ aggregation system, as follows.

### Oscillation related to the inflammation parameters

To investigate the effect of coupling-related parameters on the kinetic of systems, we varied the three parameters ${steep}$, ${infl\_ref}$ and ${k}_{{infl}}$ over a reasonably broad range, and simulated the system kinetics. A rich diversity in the kinetic behavior of the system was observed over the studied region of this three-parameter space, including monotonic kinetics, overdamped, damped, and steady oscillations. Parts of our results are shown in Fig. 3a as a contour map corresponding to Aβ monomer concentration (*z*-axis, represented as colors) as a function of time (the inner *x*-axis) with respect to a range of parameter values (${steep}$ and ${infl\_ref}$, respectively, as the outer and inner *y*-axis and ${k}_{{infl}}$, as the outer *x*-axis). In the contour map, the flat colors signify monotonic behavior, the few ridges ending in flat colors imply overdamped or damped oscillation, while consecutive ridges signify steady oscillation. In general, the most pronounced steady oscillations were observed when the steep was 20 or higher, the ${k}_{{infl}}$ was 0.001, and the ${infl\_ref}$ varied over 0.002–0.2 (the four top-left subfigures). For example, the top left subfigure with ${steep}$ value of 50 shows a curving of the ridges forming a ripple pattern, indicating that the period of oscillation is sensitive to the ${infl\_ref}$ parameter varying along the *y*-axis. Furthermore, the period of oscillation appears not to be constant along the same trajectory but reduces as time progresses.

To better characterize the effect of these coupling-related parameters on the properties of concentration oscillations, we focused on the later part of trajectories and monitored the effect of these three parameters separately, as follows:

### The ${{\boldsymbol{k}}}_{{\boldsymbol{infl}}}$ parameter

The ${k}_{{infl}}$ was varied over the range $0.1-6\times {10}^{-3}$ while maintaining fixed values for ${steep}$ and ${infl\_ref}$ at 50 and 0.005, respectively. As shown in Fig. 3a, b, the period of concentration-oscillations reduced from about 7000 s at ${k}_{{infl}}$ of $0.1\times {10}^{-3}$ to about 2000 s at ${k}_{{infl}}$ of $3\times {10}^{-3}$, before losing the regular oscillatory behavior at higher ${k}_{{infl}}$ values. Interestingly, the normalized amplitude of oscillations in concentrations (normalized concerning average concentration) showed a similar behavior decreasing in an inverse sigmoidal manner from the maximum possible value of 2 at ${k}_{{infl}}$ of $0.1\times {10}^{-3}$ to negligibly small values at ${k}_{{infl}}$ of $3\times {10}^{-3}$. Overall, a gradual shift from large-amplitude slow oscillations towards large-amplitude fast and finally small-amplitude fast oscillations were observed when ${k}_{{infl}}$ varied between $0.1\times {10}^{-3}$ and $3\times {10}^{-3}$. The regular oscillatory behavior was lost at ${k}_{{infl}}$ above $3\times {10}^{-3}$.

### The ***steep*** parameter

The oscillatory potential of ${steep}$ parameter was investigated by keeping the values of ${infl\_ref}$ and ${k}_{{infl}}$ fixed at 0.005 and $3\times {10}^{-3}$, respectively. The ${steep}$ parameter showed oscillatory behavior above the value of 20 and reached the maximum amplitude by the value of 30 and above. The regular period exhibited a slight dependence on ${steep}$ value, increasing from about 4500 s at ${steep}$ of 20 to the saturating value of around 5500 s at ${steep}$ of 100 (Supplementary Fig. 1). Overall, a shift from smaller-amplitude faster oscillations to larger-amplitude slower oscillation was observed over the ${steep}$ values of 20 − 30.

### The ***infl****_****ref*** parameter

The same analysis was carried out for ${infl\_ref}$ by keeping the values of ${steep}$ and ${k}_{{infl}}$ fixed at 50 and $1\times {10}^{-3}$, respectively. The regular period of oscillation reduced from around 8000 s to about 4000 s as the ${infl\_ref}$ varied from 0.001 to 0.1. The steady amplitude dropped steeply from maximum values of 2 to zero as ${infl\_ref}$ approached 0.1 (Supplementary Fig. 2). The system entered a non-oscillatory regime at ${infl\_ref}$ above 0.1. Overall, a shift from large-amplitude slow oscillations to small-amplitude fast oscillations was observed when the ${infl\_ref}$ parameter increased from 0.001 to 0.1. This effect is similar to that of ${k}_{{infl}}$ but opposite to that of ${steep}$ parameter.

### Oscillations related to generation and degradation parameters

The above analysis showed the propensity of system to exhibit steady oscillation within specific regions of the coupling-related parameter sub-space. To examine the effect of parameters related to generation and clearance, namely ${k}_{+}$ and ${k}_{-}$ on the kinetic behavior of system, we fixed the coupling-related parameters ${steep}$, ${infl\_ref}$ and ${k}_{{infl}}$ at 50, 0.01, and 0.003, respectively, where the system does not show an oscillatory kinetics with the so-called standard values of ${k}_{+}$ and ${k}_{-}$ (Table 1). Then evaluated the effect of variation of these two parameters, as follows:

### The ${{\boldsymbol{k}}}_{+}$ parameter

This parameter represents the rate of Aβ monomer generation, supposedly decreasing from an intrinsic value ${k}_{+0}$ at zero inflammation to a value of ${k}_{+\infty }$ at infinite inflammation. Several familial AD-related mutations are known to increase the rate of Aβ monomer generation, e.g., by favoring the amyloidogenic pathway in the proteolytic cleavage of APP^9^. It is therefore interesting to evaluate its effect on the kinetics of coupled aggregation-inflammation system. To this end, we varied ${k}_{+0}$ in the range 10^−12^ to 10^−^^8^ (the standard value of ${k}_{+0}$ was 10^−^^9^) while keeping ${k}_{+\infty }=0$. Within the range of $2\times {10}^{-10}$ till $6\times {10}^{-9}$, the system showed steady oscillations with period of ~ 2500 s and maximum normalized amplitude of up to 1.5. Interestingly, however, the system entered non-oscillatory regime at $6\times {10}^{-9}$ (Fig. 4a, b).

### The ***k****_-* parameter

This parameter represents the clearance or degradation rate of Aβ monomers directly (and Aβ oligomers and fibrils indirectly after the application of a scaling factor), which we suppose it increases from ${k}_{-0}$ at zero inflammation to ${k}_{-\infty }$ at infinite inflammation. Several lines of evidence point to the altered rate of protein degradation in neurodegenerative diseases, e.g., as a consequence of disease-related mutations or posttranslational modifications^35,36^. To evaluate the effect of variation in this parameter, we fixed the above ${k}_{+0}$ and ${k}_{+\infty }$ values, respectively at $7\times {10}^{-12}$ and 0, where the system was outside but not far from the oscillatory regime, then varied ${k}_{-0}$ in the range from 10^−6^ to $5\times {10}^{-4}$ (the standard value of ${k}_{-0}$ was $2.78\times {10}^{-4}$, and kept ${k}_{-\infty }=5{k}_{-0}$. Despite this large variation, the system did not exhibit steady oscillations (Supplementary Fig. 3). Thus, the effect of Aβ monomer degradation rate on the bifurcation of the system is in clear contrast with the effect of its generation rate (see below for the effect of degradation rate for Aβ oligomers and fibrils).

### Oscillations related to oligomerization and reverse-oligomerization processes

Subsequently, we investigated the effect of changes in aggregation-related parameters on the kinetics of the system. We started with the parameters ${j}_{1}$ and ${j}_{-1}$, which control the oligomerization and reverse-oligomerization process during primary nucleation. Several AD-related mutations and posttranslational modifications of Aβ are known to enhance Aβ oligomerization^37,38^ and alter the thermodynamic and kinetic stability of its aggregates^39,40^. In addition, anti-AD antibodies and drug candidates are often targeted at the oligomerization process and modulate these rates^41^. To simulate the effect of changes in these parameters, we fixed the values of coupling-related parameters at the level described in the “The *k_-* parameter” section and allowed generation and degradation rates to follow their assumed inflammation dependence. We then varied ${j}_{1}$ in steps from 10^−^^8^ to 10^−^^6^ (the standard value of ${j}_{1}$ was $6.7\times {10}^{-8}$), assuming that this parameter itself was inflammation-independent, i.e., ${j}_{10}={j}_{1\infty }={j}_{1}$. The Aβ aggregation system started showing oscillatory behavior from the ${j}_{1}$ value of 10^−^^7^ and reached a steady oscillation of about 2500 s period and 1.5 normalized amplitude (Fig. 4c, d). The system retained its oscillatory kinetics till ${j}_{1}={10}^{-6}$.

A similar analysis was performed for the reverse-oligomerization rate ${j}_{-1}$, by varying ${j}_{-1}$ from 10^−^^5^ to 2 × 10^−^^3^ in steps, while keeping ${j}_{1}=6.7\times {10}^{-8}$. The system showed relatively weak oscillatory behavior between ${j}_{-1}$ of 10^−^^3^ and $3\times {10}^{-3}$, having a maximum normalized amplitude of 0.8 and showing a decrease in the period from 2000 s to 1000 s (Supplementary Fig. 4).

### Oscillations related to fibrillation and elongation processes

In our model, the fibrils grow by primary or secondary nucleation processes, followed by elongation. The effects of the kinetic parameters related to fibrillation were studied after fixing the coupling-related parameters as described above and setting ${j}_{1}=6.7\times {10}^{-8}$ and ${j}_{-1}=9.7\times {10}^{-5}$. With these starting parameters the system shows mild oscillation with a period of about 1940 s and a normalized amplitude of 0.342. We varied the rate constants ${j}_{2}$ (related to oligomer conversion) from 10^−^^9^ to 10^−^^11^, ${j}_{3}$ (related to fibril elongation) from 10^5^ to 10^7^, and ${j}_{4}$ (related to secondary nucleation) from 2 to 200 in steps. In our model, all these steps are irreversible, that is, ${j}_{-2}={j}_{-3}={j}_{-4}=0$. No considerable change in the mild oscillatory behavior of the system was observed in this rather broad range of values for fibrillation-related parameters (Supplementary Fig. 5).

### Oscillations related to the clearance of Aβ aggregates

Aβ oligomers and fibrils are potentially cleared by the recruited macrophages and to a lower extent, activated microglia via degradation mechanism over and above proteasomal degradation pathways^42^. Mutations and modifications in the Aβ sequence may alter the stability of its aggregates against proteolytic pathways^35^. Besides, new therapeutic interventions, including monoclonal antibodies, modify the kinetics of clearance of the Aβ aggregates^43,44^. In our model, the clearance kinetics of oligomers and fibrils is described by the degradation rate ${k}_{-}$, scaled down by constant factors ${\gamma }_{o}$ for oligomers and ${\gamma }_{f}$ for fibrils. To investigate the effect of oligomer clearance rate on the system kinetics, we varied the parameter ${\gamma }_{o}$ from 0.01 (corresponding to highly stable oligomers against proteolytic degradation, ${k}_{-}$ is attenuated to 1%) to 1.0 (corresponding to highly susceptible oligomers to proteolytic degradation, or when clearing is enhanced by therapy) in steps. Interestingly, the system shows weak but steady oscillation between the values of ${\gamma }_{o}$ = 0.01–0.2, with the maximum normalized amplitude of 0.6 and period reducing from about 2000 s to about 1000 s with increasing value of ${\gamma }_{o}$ (Fig. 4e, f). The system becomes non-oscillatory above ${\gamma }_{o}=0.2$ and remains so till the value of 1.0.

Subsequently, we examined the effect of fibrillar clearance rate by setting ${\gamma }_{o}=0.05$, such that the system shows mild oscillation with a period of about 1940 s and normalized amplitude of 0.342 (as in the “Oscillations related to fibrillation and elongation processes” section). The attenuation factor of fibril clearance, ${\gamma }_{f}$ was then varied between 0.01 (corresponding to highly stable fibrils against proteolysis) to 1 (corresponding to highly susceptible fibrils to proteolysis) in steps. Remarkably, this variation had no considerable effect on the oscillatory behavior of the system (Supplementary Fig. 6).

## Discussion

Here, we have presented a minimal model of a coupled aggregation-inflammation system for Aβ peptide, in which Aβ monomers proceed toward aggregation through primary and secondary nucleation and elongation processes, the generated Aβ oligomers and fibrils induce inflammation, and the inflammation in turn enhances Aβ clearance and reduces its generation (Fig. 1). Despite its simplicity, the model exhibits remarkably rich dynamics, especially a large propensity for oscillatory kinetics depending on the parameters controlling the coupling between aggregation and inflammation (Fig. 3). Several Aβ aggregation-related parameters, esp. rates of generation and clearance of Aβ monomers (${k}_{+}$ and ${k}_{-}$), rate of Aβ oligomerization during primary nucleation (${j}_{1}$), and the parameter governing the stability of Aβ oligomers against proteolytic degradation (${\gamma }_{o}$), are shown to modulate the oscillatory regime of system dynamics (Fig. 4).

Biochemical oscillations are rather common and occur in a broad range of cellular biological contexts underlying circadian rhythms, DNA synthesis and mitosis, development and so on^45^. The main requirements for (bio)chemical oscillators are negative feedback loops accompanied by implicit or explicit time delays and a high level of nonlinearity in system equations (often involving interaction between chemical species), which are not uncommon to be fulfilled in biochemical systems^45^. The oscillatory response of the immune system to antigenic stimulation has been reported decades ago^46^ and a growing body of evidence during past decades has demonstrated that many aspects of the immune response follow oscillatory kinetics^47^. It is well known that Aβ aggregates especially oligomers can trigger a cascade of inflammatory response by activating microglia and astrocytes, involving recruitment of peripheral macrophages to the sites of Aβ deposition and promoting removal of Aβ aggregates by recruited macrophages and, to a lower extent, activated microglia^13^. In addition, the temporally regulated emergence of pro- and anti-inflammatory microglia and macrophages and the corresponding cytokines control the temporal evolution of the immune response, leading to the end of the immune response after re-establishing the homeostatic state without Aβ aggregates^13,19^. Unlike several published models of the immune system of AD^47–50^, the minimal model presented here does not attempt to capture the complex internal dynamics of the immune response to Aβ aggregates, instead, represents the generated inflammation simply as a time-dependent state variable, ${infl}$, capable of modulating the production and clearance rates of Aβ, respectively ${k}_{+}$ and ${k}_{-}$, according to a Hill-type response function (${resp}$). In coupling between inflammation and Aβ system, the model is based on two simple premises, that inflammation promotes clearance of Aβ, as supported by a vast body of experimental data^11,13,18^, and that inflammation suppresses Aβ generation. The latter premise admittedly goes against reports suggesting the presence of vicious cycles of Aβ generation enhancement by inflammation^17,51^, but is supposed here to represent a physiological state of the system more robust against progression to a pathological state. Despite the very simple structure of our model, it exhibits a rich dynamical behavior depending on the coupling-related parameters, most notable a pronounced propensity for steady oscillations. The oscillatory potential of the system can be attributed to the presence of a general oscillatory motif, the negative feedback with time delay introduced by a series of intermediate steps between Aβ monomer and inflammation^45^. As expected, the emergence of significant oscillations depended on appropriate time constants underlying the production and consumption of various Aβ species and inflammation. Interestingly, the periodicity and relative amplitude of oscillations depended on the coupling-related parameters, e.g., within the oscillatory basin of parameter space, an increase in the parameter ${k}_{{infl}}$ representing higher tendency of inflammation response to control itself led to a gradual shift from large-amplitude slow oscillations to small-amplitude fast oscillations. A similar trend was observed for the parameter ${infl\_ref}$ representing the general sensitivity of the coupled system to inflammation levels, while the parameter ${steep}$ representing the steepness of the system response to inflammation had an opposite effect.

In relation with the above-mentioned coupling-related parameters, many molecular factors are naturally involved in how the Aβ clearance pathways (e.g., in macrophages, microglia, or in relation to transport to blood or CSF) and Aβ generation (by neurons and astrocytes) respond to inflammation and how the inflammation response governs its self-limiting kinetics (e.g., by a variety of pro- and anti-inflammatory macrophages and microglia and the corresponding cytokines)^11,13,15,16^. Consequently, alterations in these molecular factors have the potential to push the system away from its homeostatic state towards oscillatory kinetics. Besides, our data shows that the AD-related mutations and posttranslational modifications which affect the intrinsic rates of Aβ generation (through parameter ${k}_{+}$), Aβ oligomerization (through parameter ${j}_{1}$), and Aβ clearance (through a parameter ${k}_{-}$), esp. clearance of Aβ oligomers (through a parameter ${\gamma }_{o}$), shift the oscillatory regime and induce oscillatory kinetics at normally non-oscillatory region of coupling-related parameter space. Therefore, a complex interplay of molecular factors related to Aβ aggregation, inflammation and coupling between them seem to underlie the rich dynamics of the system, including its pronounced propensity for steady oscillations. Notably, rich dynamics of protein aggregation systems have previously been modeled based on the coupling between Aβ and tau protein aggregation in AD^52^, or based on the coexistence of kinetically distinct aggregates in prion systems^53^. Based on our data, we argue that such steady oscillations could lead to immune system exhaustion over prolonged periods and eventually lead to the failure of compensatory mechanisms, hence contributing to a transition to a more progressive phase of amyloid pathology in pre-clinical AD. Accordingly, we propose that detection of such oscillations in levels of Aβ species or neuroinflammation could be considered as potential biomarkers for the early diagnosis of AD in normal, mild cognitive impairment or preclinical AD cases, especially considering the relatively long periodicities (in the order of tens of minutes to few hours) and potentially large amplitude of oscillations shown here.

The complex interplay between cancer and the immune system has been the subject of extensive studies in the past decades and oscillations in cancer pathology (remission–recurrence) or inflammation have been predicted and experimentally detected^54,55^. In AD, the altered level and kinetic of systemic and neuroinflammation are shown by numerous reports based on blood (plasma) and CSF markers of inflammation and neuroimaging methods^56–61^, however, to our best knowledge, there is no report of experimental detection of oscillatory dynamics in different stages of AD. Our data indicate the potential presence of oscillations in amyloid pathology or inflammation in AD and propose that with vigilant monitoring of related biomarkers at proper time intervals such oscillations could be detected. Notably, several neuro-imaging techniques (including PET, MRS, and fMRI) are available to monitor neuroinflammation and amyloid pathology at sufficiently short time intervals^59–62^, and some neuroimaging observations already point to an oscillating amyloid burden in a mouse model of AD^63^. Furthermore, oscillations in the coupled Aβ aggregation and inflammation system may be related to perturbations in circadian rhythms and sleep disturbances frequently associated with pre-clinical and early stages of AD^64^. We, therefore, propose that the search for oscillatory dynamics in AD be included in epidemiological studies that search for early biomarkers of AD.

There is currently no cure for AD, but palliative treatment. An active approach in searching for efficient anti-AD treatment is to develop agents targeting Aβ aggregation at different steps, e.g., reducing its generation, inhibiting its aggregation, or promoting its clearance^44^. The results presented here based on a minimal model of coupled aggregation-inflammation show the potential of such interventions in profoundly altering the kinetics of the system, e.g., inducing oscillations, in the in vivo context. It is not clear what the effect of such oscillatory kinetics would be on the outcome of therapeutic interventions, but we suggest that this hitherto largely neglected aspect should be taken into consideration in clinical trials of potential anti-AD drug candidates. The presence and characteristic features of oscillations in treated and non-treated groups could then provide additional information on the efficacy of anti-AD agents and facilitate the interpretation of trial results. Finally, it is worth mentioning that the mutual interplay between a pathological process and the immune system is a rather generic phenomenon and complex dynamical patterns could emerge in a broad range of pathophysiological systems. The modeling approach presented here is therefore potentially applicable to many such systems, including non-AD neurodegenerative and other diseases.

To summarize, we introduce a minimal model of AD-related Aβ aggregation coupled to inflammation, based on a well-validated model of Aβ aggregation in vitro. The simulated behavior of this simple model in dependence of various parameters related to Aβ aggregation and the coupling between inflammation and Aβ aggregation demonstrates the rich dynamics of this system, including a pronounced propensity to steady oscillations. We search for regions of coupling-related parameter space in which a drastic shift in system dynamics towards oscillatory dynamics, i.e., bifurcation, occurs and demonstrate how changes in Aβ aggregation-related parameters can shift such bifurcations. Our data provides a simple mechanism for potential steady oscillations in Aβ and inflammation levels and suggests that such steady oscillations could lead to eventual exhaustion of the immune system and failure of compensatory mechanisms over prolonged periods and contribute to the transition of AD into a more progressive amyloid pathology phase. Furthermore, we propose that the presence and features of such oscillations should be taken into account in the search for early biomarkers of AD and potential drugs in anti-AD clinical trials.

## Methods

The system was modeled by monitoring the species concentrations using a system of coupled ODEs. Our model describes the early phase of AD when there is no significant build-up of plaques. Therefore, in our model, the brain is considered a homogeneous compartment of fixed volume with respect to the concentrations of Aβ aggregation species. In our simulations the Aβ monomer concentration is 0 at $t=0$, however, we have checked the validity of the results starting from initial Aβ monomer concentrations at the physiologically relevant levels (Supplementary Fig. 7).

We translated the equations into *Mathematica* notebooks (version 12.3.1.0)^65,66^ and used the numerical differential equation solver (*NDSolve*) to generate an interpolating function for the system that approximates the behavior within the boundaries of 0–10^7^ s (115 days, 17 h, and 47 min). We have used the automatic option of the *NDSolve* for the adaptive procedure to determine the step size and number of steps to satisfy the default *AccuracyGoal* and *PrecisionGoal* parameters. In standard workstations (AMD Ryzen 7 2700, eight-core 3.2 GHz processor, 64 GB RAM) the generation and plotting of the time-course in the *Mathematica* notebook required about 10 min with marginal improvement upon parallelization of *NDSolve*.

To calculate the aggregation rates and lag times for systems with various initial monomer concentrations (as shown in Fig. 2e), we used a logistic function for the fitting process (Least Square Method, SciPy). The growth constant in the logistic function corresponds to the aggregation rate and the *t*-axis intercept with the tangent at the inflection point gives us the lag time.

Time course simulations were performed till 10^7^ s at 1 s time intervals using the deterministic numerical method of lines algorithm. The early behavior of the model was established from the knowledge of the steady-state concentrations of the Aβ species and the reported kinetics of the aggregation process. The *FindPeaks* function within *Mathematica* was utilized to measure the peak-to-peak distance as period (Supplementary Fig. 8). The baseline in the latter part of the steady oscillation or the damped oscillation at 50,000 s was determined in a way that the mid-point between the valleys and troughs was one (Supplementary Fig. 9a). The amplitudes were normalized with respect to the baseline (Supplementary Fig. 9b). The three-dimensional surfaces of the contour maps were plotted with the *ArrayPlot* function in *Mathematica* with the default density gradient color function.

The system contained five state variables, ${a}_{m}$, ${a}_{o}$, ${a}_{{fp}}$, and ${a}_{f}$, respectively, representing concentrations of the monomer, oligomer, fibril particles, and fibrils of Aβ, and ${infl}$, representing the inflammation level. The dynamics of the system were modeled using a system of coupled ODE (Eqs. 1–5). The inflammation level ${infl}$ was calculated using a differential equation that depends on the concentrations and the inflammatory potentials of different aggregation states of Aβ (Eq. 5). We have used a numerical differential equation solver (*NDSolve*) within *Mathematica* for the time course of 10^7^ s with *MaxSteps* = 10^8^. The value of *MaxSteps* was a balance between the accuracy and compilation time of the *Mathematica* notebook. We have generated the time course for each situation with differing values of the parameter under investigation using *ParallelTable*, which improves the compilation time.

### The closed uncoupled system

To simulate the closed uncoupled system, the rate of generation of Aβ monomers and the rate of degradation of Aβ monomers were fixed at 0, ${k}_{+}={k}_{-}=0$. The initial Aβ monomer concentration was varied from 0.01 μM, 0.02 μM, 0.05 μM, 0.1 μM, 0.2 μM, 0.5 μM, 1.0 μM, 2.0 μM, 5.0 μM, 10.0 μM, 20.0 μM, 50 μM, and 100 μM, while the initial Aβ oligomer (${a}_{o}$), fibril particle (${a}_{{fp}}$) and fibril (${a}_{f}$) concentrations were set to 0. The initial level of inflammation was set to ${infl}=0$, and the inflammatory potential of the aggregation species was set to ${\delta }_{o}={\delta }_{f}=0$. All other parameters are as described in Table 1.

### The open uncoupled system

In the open uncoupled system, the rate of generation of Aβ monomers was set to ${k}_{+}={10}^{-12},$ and the rate of Aβ clearance was set to ${k}_{-}=2.78\times {10}^{-5}$ (Table 1 and Fig. 2f). The choice of these parameter values was supported by previous reports and validated based on the steady-state concentration of different Aβ species. The simulation was started with the concentration of Aβ monomer, oligomer, fibril particle and fibrils set to 0. The initial level of inflammation was set to ${infl}=0$, and the inflammatory potential of the aggregation species was set to ${\delta }_{o}={\delta }_{f}=0$. The use of a second set of values for generation and clearance rates (${k}_{+}={7.34\times 10}^{-12}$ and ${k}_{-}={2.78\times 10}^{-4}$) did not lead to a significant difference in the steady state (Fig. 2f).

### The open-coupled system

The system was coupled with the inflammation by setting the inflammatory potentials of the oligomer to ${\delta }_{o}=800,000$ and of the fibril to ${\delta }_{f}=200,000$, reflecting the higher potential of Aβ oligomers than fibrils to induce inflammation. In response to generated inflammation, the rates of Aβ monomer generation (${k}_{+}$) and degradation (${k}_{-}$) were varied between their intrinsic values (${k}_{+0}$ and ${k}_{-0}$) and post-inflammation values (${k}_{+\infty }$ and ${k}_{-\infty }$) according to a Hill-type response function (see Eqs. 6 and 7). The response function (${resp}$) varied between 0 and 1. The simulation was started with the concentration of Aβ monomer, oligomer, fibril particle, and fibrils set to 0. The initial level of inflammation was set to ${infl}=0$. All other parameters are as shown in Table 1. All subsequent calculations were performed in the open-coupled system.

### Test for convergence of the open-coupled system

We have checked the convergence properties of the equations by fixing the initial concentrations of the Aβ monomers to ${a}_{m}={0\;\mu {\mathrm{M}},0}.\,3\;\mu {\mathrm{M}},\,0.6\;\mu {\mathrm{M}},\,0.9\;\mu {\mathrm{M}},\;{\rm{and}}\; 1.2\;\mu {\mathrm{M}}$. Supplementary Fig. 7 shows the phase space of the Aβ oligomer, ${a}_{o}$ vs Aβ monomer, ${a}_{m}$, converging to an overlapped limit cycle—showing convergence to steady oscillation. The convergence was achieved within $5\times {10}^{4}$ s, within 0.5% of the time course. The phase space was drawn using *ParametricPlot* in *Mathematica* with *MaxRecursion* = *5*. The *MaxRecursion* is a balance between the accuracy and compilation time of the *Mathematica* notebooks.

### Exploring the parameter space

The role of the parameters was explored individually by keeping all the other parameters fixed. The parameter under investigation was varied in a linear array consisting of 200 or more values. The range of the parameter was within one order of magnitude, that is, 10-fold less to 10-fold more than the base value reported in Table 1. We have checked the parameters ${steep}$, ${infl\_ref}$, ${k}_{{infl}}$, ${k}_{+0}$ (with ${k}_{+\infty }=0$), ${k}_{-0}$ (with ${k}_{-\infty }=5{k}_{-0}$), ${j}_{1}$, ${j}_{-1}$, ${j}_{2},\,{j}_{3}$, ${j}_{4}$, ${\gamma }_{o}$ and ${\gamma }_{f}$ (see Eqs. 1–6 and Table 1 for the definition of parameters). We investigated each parameter within individual notebooks, which usually took approximately 10 min to compile in standard workstations (AMD Ryzen 7 2700, eight-core 3.2 GHz processor, 64 GB RAM).

We inspected the time courses of concentrations of Aβ aggregation species by plotting the time course of 10^5^ s for regular intervals of each parameter under investigation. For example, we inspected 25 uniformly distributed plots where we changed the parameter under investigation in 100 equal steps. The most visible dynamical profiles could be seen in the case of Aβ monomer concentration ${a}_{m}$, while other Aβ species and inflammation levels showed similar albeit less clear profiles. The results have therefore been shown mainly on the Aβ monomer concentrations.

The contour maps were created using *ArrayPlot* by keeping the time along the *x*-axis, and the parameter under investigation along the *y*-axis, both in linear scale. The *z*-axis of the plots showed the concentration of the Aβ monomer. The contour maps were drawn after the initial spike in concentration to identify the finer features of the stabilized oscillation after 5000 s or more, using the *ColorFunction “M10DefaultDensityGradient”* in *Mathematica*.

We plotted the normalized amplitude of steady oscillation with the parameter under investigation and the wave period of steady oscillation with the same parameter. These pairs of plots show the bifurcation properties of the individual parameters. The trends of the bifurcation properties were compatible between the pairs of plots.

#### Effect of the inflammation-related parameters

The ${steep}$ parameter was varied from 0.5 to 100 in 200 steps. The ${k}_{{infl}}$ parameter was varied from 0.00005 to 0.01 in 200 steps. The ${infl\_ref}$ parameter was varied from 0.001 to 0.5 in 500 steps.

#### Effect of the rate of Aβ monomer generation

The ${k}_{+0}$ parameter was varied from 10^−13^ to 10^−^^11^ in 100 steps and 10^−^^10^ to 10^−^^8^ in 100 steps, while ${k}_{+\infty }=0$.

#### Effect of the rate of Aβ monomer degradation

The ${k}_{-0}$ parameter was varied from 10^−^^6^ to 5 × 10^−^^4^ in 500 steps. The ${k}_{-\infty }$ parameter was varied linearly with ${k}_{-0}$, with ${k}_{-\infty }=5\times {k}_{-0}$.

#### Effect of the rate of Aβ oligomer formation during primary nucleation

The ${j}_{1}$ parameter was varied from 10^−^^10^ to 10^−^^8^ in 100 steps and 10^−^^8^ to 10^−^^6^ in 100 steps. Effect of the rate of Aβ disaggregation, oligomer dissociation to monomer: The ${j}_{-1}$ parameter was varied from 10^−^^10^ to 10^−^^8^ in 100 steps and 10^−^^8^ to 10^−^^6^ in 100 steps.

#### Effect of the rate of irreversible Aβ oligomer conversion

The ${j}_{2}$ parameter was varied from 10^7^ to 10^9^ in 100 steps and 10^9^ to 10^11^ in 100 steps.

#### Effect the rate of irreversible Aβ fibril elongation

The ${j}_{3}$ parameter was varied from 10^5^ to 10^7^ in 100 steps and 10^7^ to 10^9^ in 100 steps.

#### Effect of the rate of irreversible Aβ oligomer formation during secondary nucleation

The ${j}_{4}$ parameter was varied from 10^−^^2^ to 1 in 100 steps and 2 to 200 in 100 steps.

#### Effect of the rate of clearance of Aβ oligomers

The ${\gamma }_{o}$ parameter was varied from 10^−^^4^ to 10^−^^2^ in 100 steps and 10^−^^2^ to 1 in 100 steps.

Effect of the rate of clearance of Aβ fibrils: The ${\gamma }_{f}$ parameter was varied from 10^−^^4^ to 10^−^^2^ in 100 steps and 10^−^^2^ to 1 in 100 steps.

In all cases, other parameters were fixed at the level shown in Table 1.

We performed the calculations in a modular manner using the notebook feature of *Mathematica*. Additional calculations were performed using COPASI^67^, as described below.

### Measuring period of oscillation

The period of the oscillations changed during the time course to asymptotic values, as seen in Supplementary Fig. 8. We have considered the period after the stabilization period of 50,000 s when the period reaches the asymptotic value for steady oscillations. The period was measured as the peak-to-peak distance in time using the function *FindPeak* within *Mathematica*. The period (and amplitude) were measured as the mean value of the last 5 peaks, which allowed us to reliably determine the period (and amplitude) of fluctuations in sustained oscillations. We have used the algorithm with maximum sensitivity, that is, with minimal Gaussian blurring for peak detection. The algorithm used the default Gaussian blurring of $\sigma ={(\frac{{Log}\left(n\right)}{{Log}\left(100\right)})}^{2}$, where *n* is the number of data points, which translates to 6.25 s for the 10^5^ data points distributed over 10^5^ s. This sensitive algorithm also detects peaks in damped oscillations. In such cases, the algorithm detects progressively smaller peaks, and consequently, the calculated average amplitude could become negligibly small, and the spacing between peaks (periods) could become irregular.

### Measuring the amplitude of oscillation and normalization

We have fixed the Aβ monomer to 0 at $t=0$. The amplitude of the initial oscillations reduced from a large fluctuation to asymptotic values, as seen in Supplementary Fig. 9a. We have determined a baseline (= 1 after normalization) as the midpoint between the peaks and troughs of steady oscillation after the stabilization period. The peak could be found using the *FindPeak* command as described in the measurement of periods. We measured the troughs by finding the peaks after reflecting the oscillations in concentration around the time axis. After normalization, the maximum possible amplitude of fluctuations can be 2. The normalized oscillations are shown in Supplementary Fig. 9b. All simulations, calculations, and analyses were performed using Mathematica (version 12.3.1.0)^66^.

### Sensitivity analysis

The software COPASI (Complex Pathway Simulator, version 4.39, Build 272)^67^ was used to calculate the system sensitivities in non-oscillatory steady-states. First, we reproduced all the time series results to ensure compatibility between numerical solutions of the system of coupled differential equations solved using Mathematica and COPASI. The COPASI results were calculated using the basal values of the parameters as tabulated in Table 1 in the main text. The scaled sensitivities of the non-constant concentrations of species due to all parameters were calculated in the time series mode after achieving non-oscillatory steady-states by 10^6^ s. The scaled sensitivity of the state variable $y$ with respect to parameter $x$ was calculated as $(\frac{\partial y}{\partial x}\times \frac{x}{y})$. In Supplementary Tables 1 and 2, we have compared the values of all the rate constants related to aggregation and inflammation but not the exponents (orders of reactions).

### Supplementary information

Supplementary Information

Supplementary Data

## Data Availability

Source data, COPASI models, and Python scripts are provided in this paper. Mathematica notebooks are available from the first author and corresponding author upon reasonable request.
