# Supplementary material for: Bifurcations in coupled amyloid-β aggregation-inflammation systems
Source: NPJ Syst Biol Appl. 2024 Jul 30;10:80. doi: 10.1038/s41540-024-00408-7 (PMC11289389; doi:10.1038/s41540-024-00408-7)
Supplement: Supplementary file 1 — Supplementary Information [file 41540_2024_408_MOESM1_ESM.pdf]

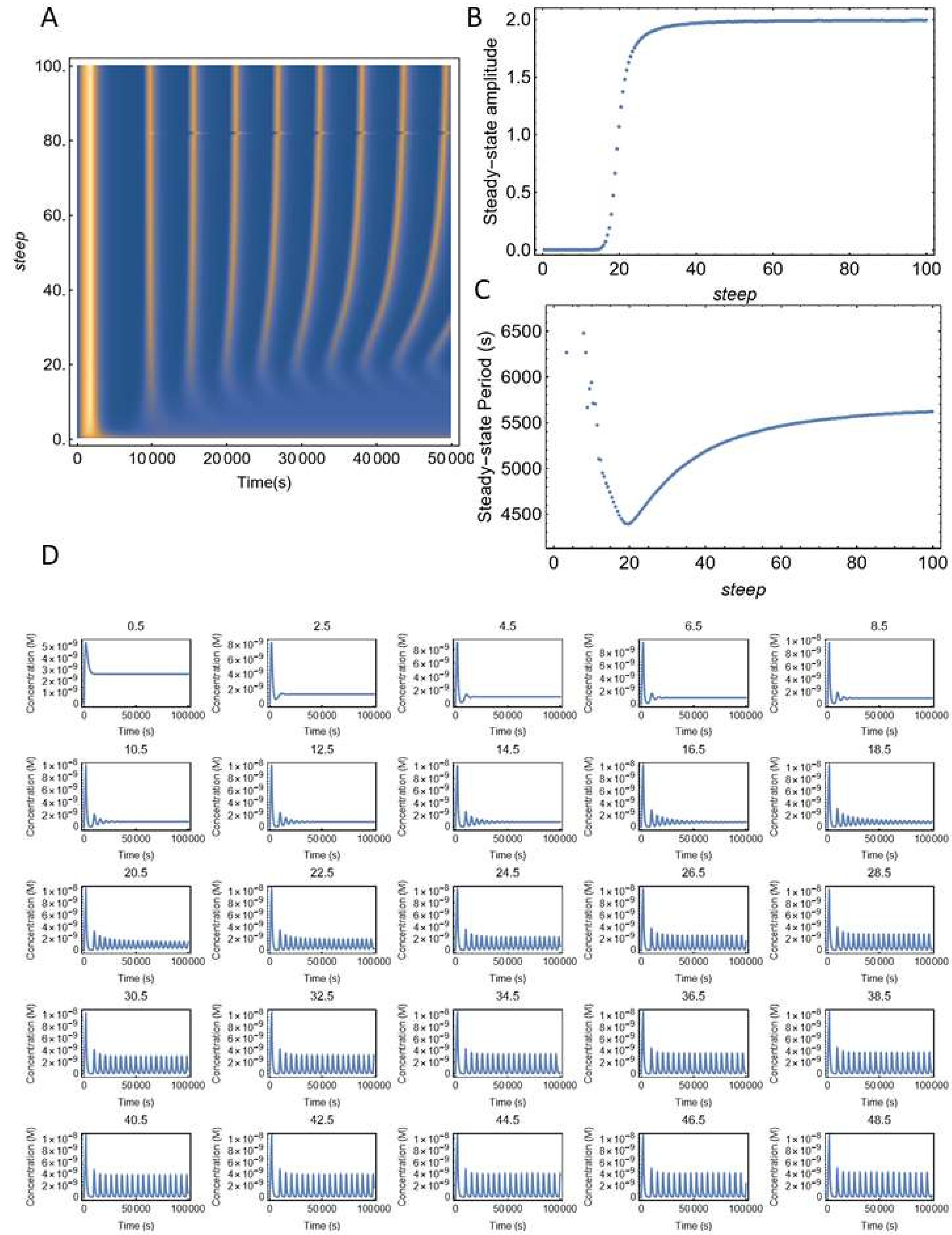

Supplementary Figure 1. The oscillatory potential of the *steep* parameter. (a) The contour map of the Aβ monomer with respect to the *steep* parameter along the y-axis and the time along x-axis. Golden color implies peak of monomer concentration (z-axis) and the blue color implies valley of Aβ monomer concentration. (b) The normalized steady-state amplitude of oscillation peaks to maximum value of 2 at the *steep* value 20. (c) The steady-state period of oscillation has a regular trend beyond the *steep* value 20. (d) the time-course of Aβ monomer concentrations for different *steep* values (mentioned on top of each panel).

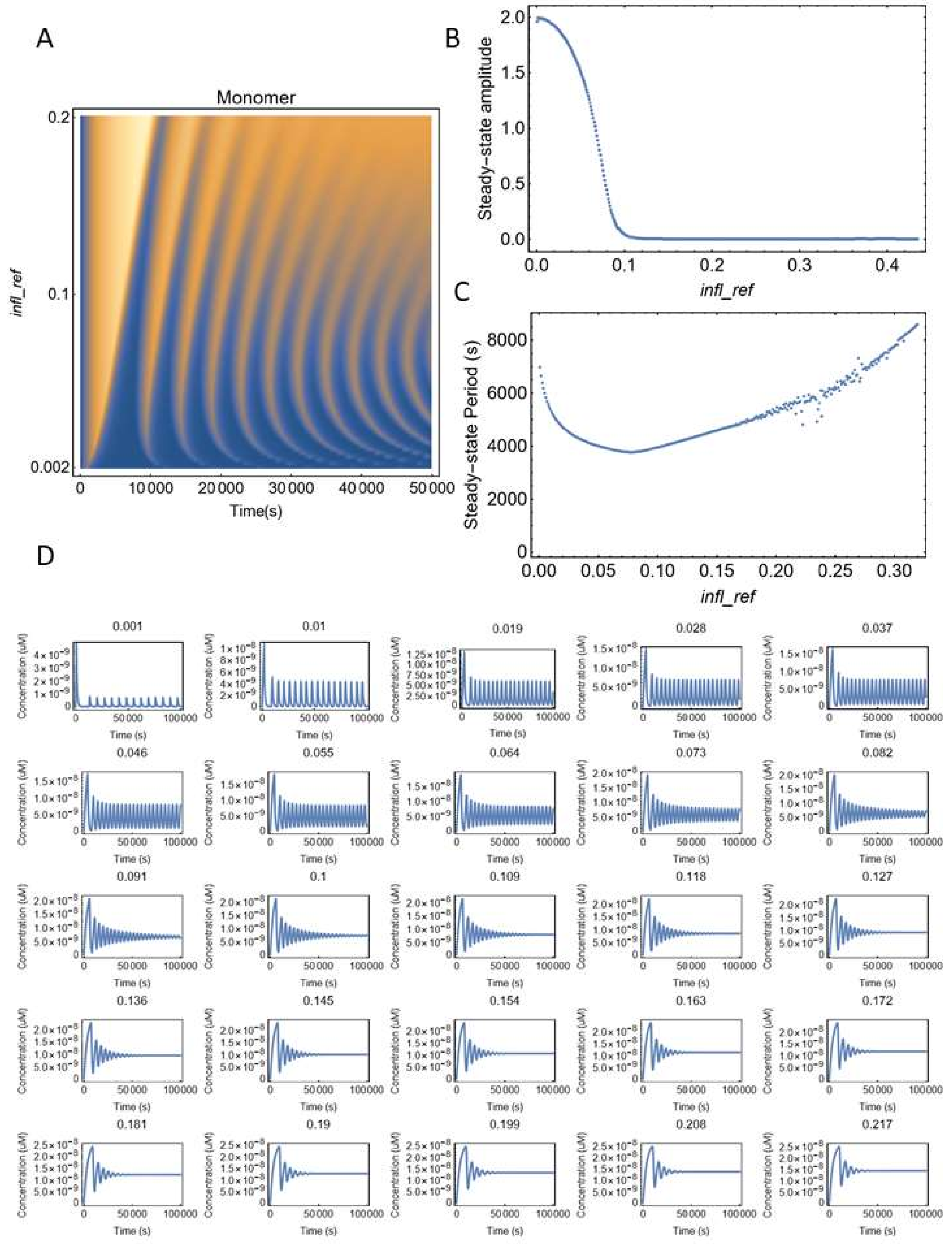

Supplementary Figure 2. The oscillatory potential of the *infl_ref* parameter. (a) The contour map of the Aβ monomer with respect to the *infl_ref* parameter along the y-axis and the time along x-axis. Golden color implies peak of monomer concentration (z-axis) and the blue color implies valley of Aβ monomer concentration. (b) The normalized steady-state amplitude of oscillation disappears at the *infl-ref* value 0.1. (c) The steady-state period of oscillation has a regular trend before the *infl_ref* value 0.1. (d) the time-course of Aβ monomer concentrations for different *infl_ref* values (mentioned on top of each panel).

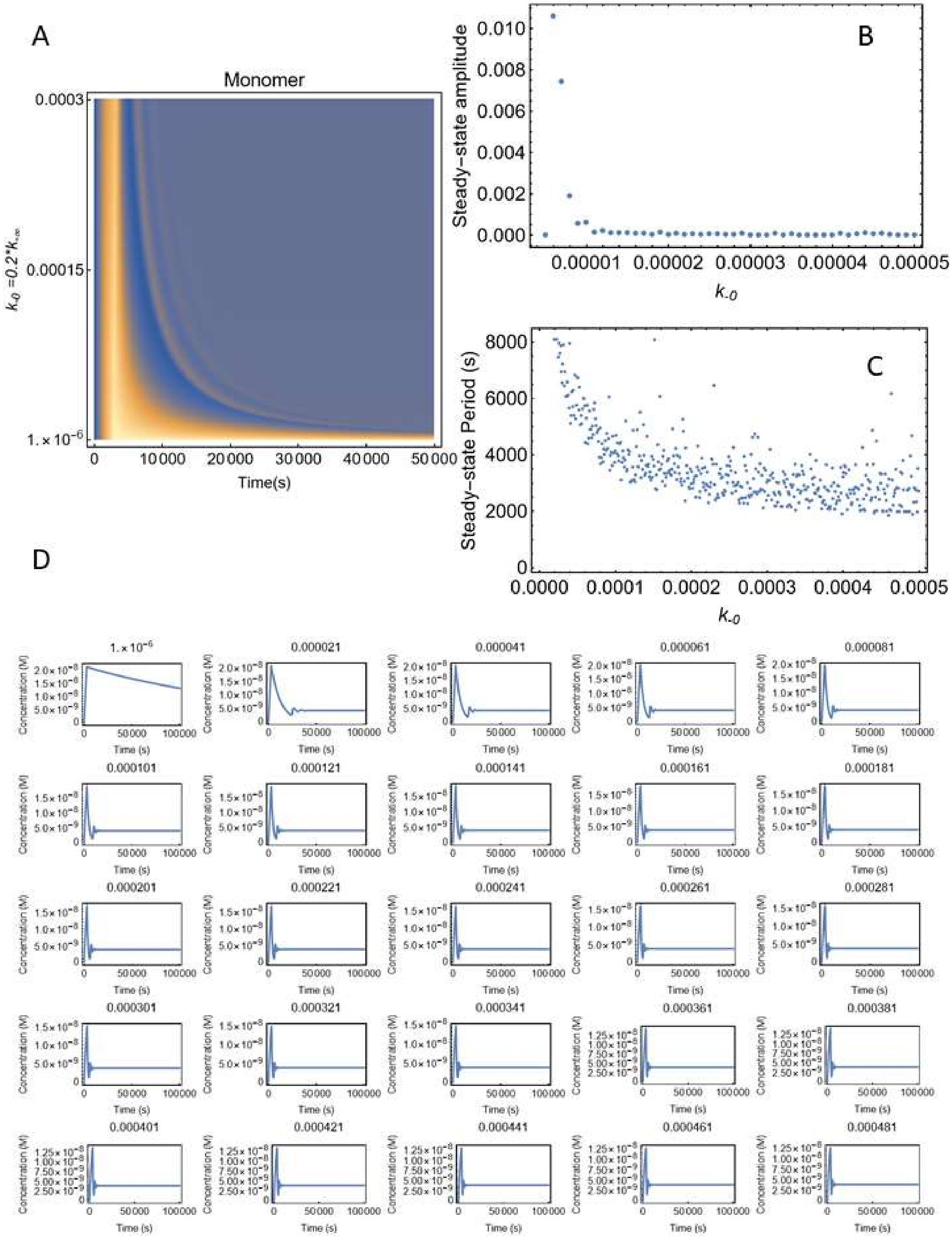

Supplementary Figure 3. The k_- parameter does not affect the oscillatory behavior. (a) The contour map of the Aβ monomer with respect to k_- parameter along the y-axis and the time along x-axis. Golden color implies peak of monomer concentration (z-axis) and the blue color implies valley of Aβ monomer concentration. (b) The normalized steady-state amplitude of oscillation is insignificant. (c) The steady-state period of oscillation shows high scatter. (d) the time-course of Aβ monomer concentrations for different k_- values (mentioned on top of each panel) does not show steady oscillation.

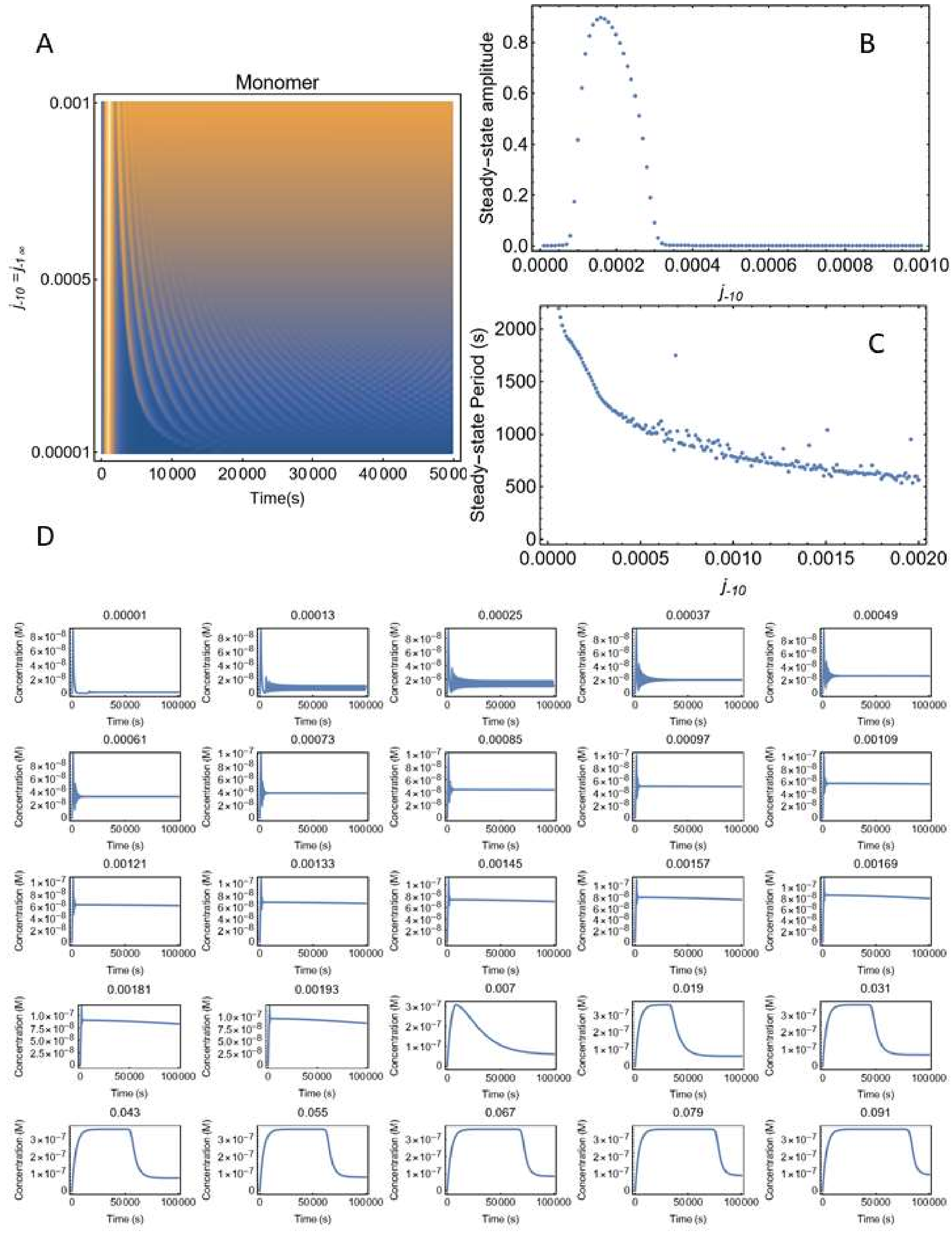

Supplementary Figure 4. The j_{-1} parameter has minor effect on the oscillatory behavior. (a) The contour map of the Aβ monomer with respect to j_{-1} parameter along the y-axis and the time along x-axis. Golden color implies the peak of monomer concentration (z-axis) and the blue color implies the valley of Aβ monomer concentration. (b) The normalized steady-state amplitude of oscillation is significant between 0.0001 and 0.0003. (c) The steady-state period of oscillation shows a regular trend till 0.0003. (d) the time-course of Aβ monomer concentrations for different j_{-1} values (mentioned on top of each panel) shows steady oscillation in a narrow range.

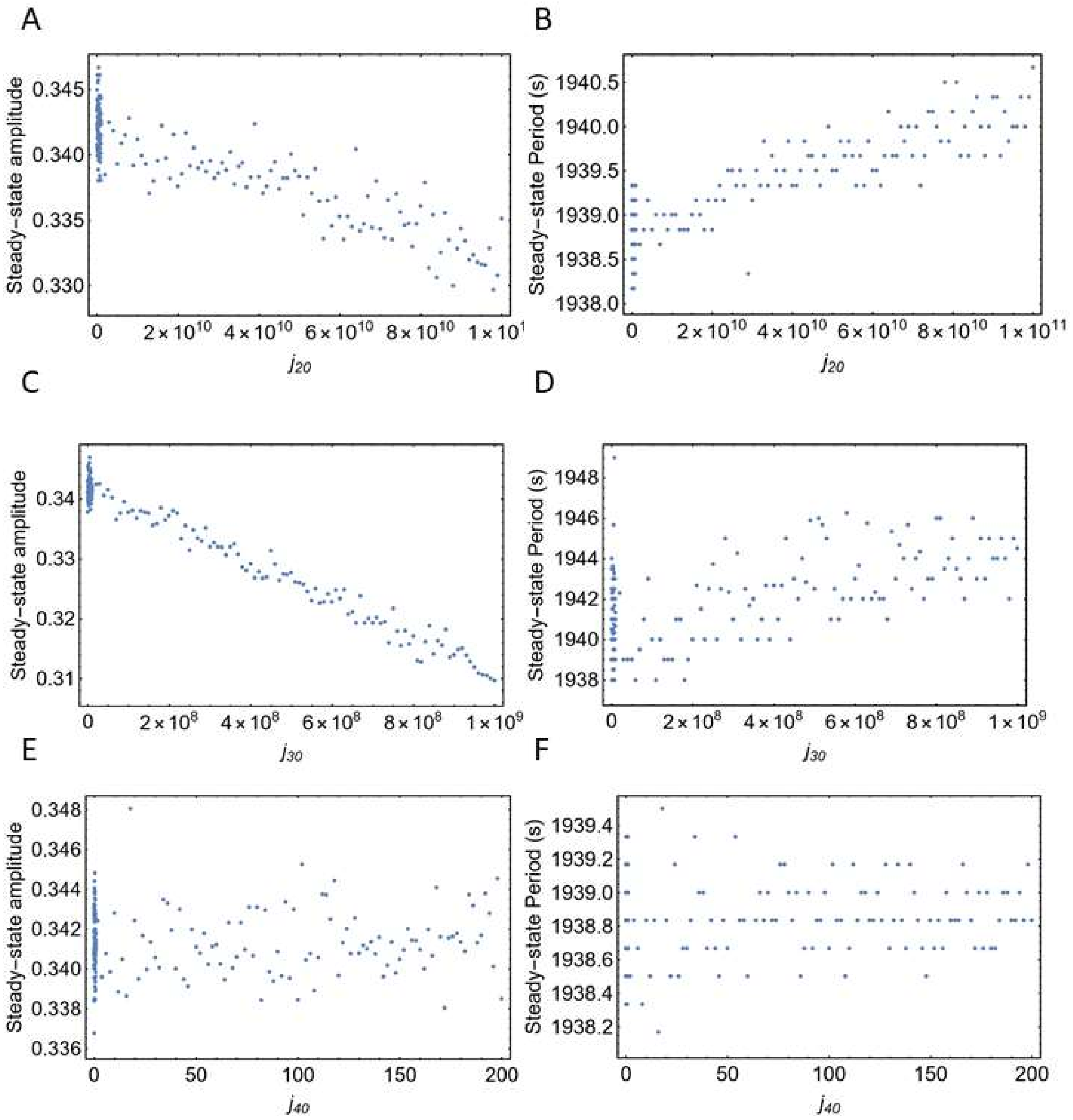

Supplementary Figure 5. The j_2 , j_3 , j_4 parameters do not affect the oscillatory behavior. (a) The normalized steady-state amplitude of oscillation of the Aβ monomer with respect to the j_2 parameter shows a high scatter. (b) The steady-state period similarly shows a high scatter for the j_2 parameter. A similar absence of steady oscillation is observed with respect to j_3 for amplitude (c) and period (d), and the same behavior is observed with respect to j_4 for amplitude (e) and (f) period.

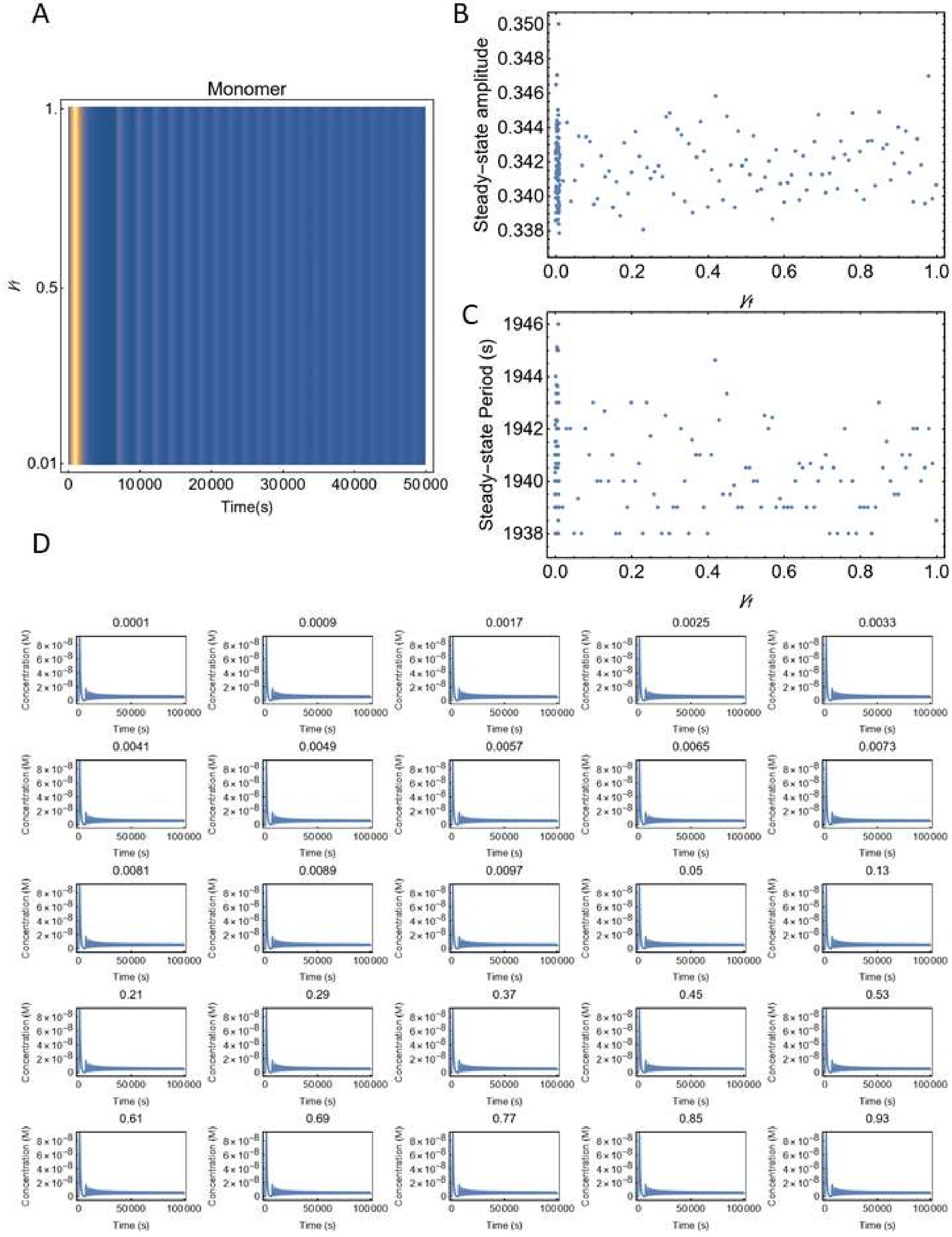

Supplementary Figure 6. The γ_f parameter does not affect the oscillatory behavior. (a) The contour map of the Aβ monomer with respect to γ_f parameter along the y-axis and the time along x-axis. The golden color implies the peak of monomer concentration (z-axis) and the blue color implies the valley of Aβ monomer concentration. (b) The normalized steady-state amplitude of oscillation is insignificant. (c) The steady-state period of oscillation shows high scatter. (d) the time courses of Aβ monomer concentrations for different γ_f values (mentioned on top of each panel) do not show steady oscillation.

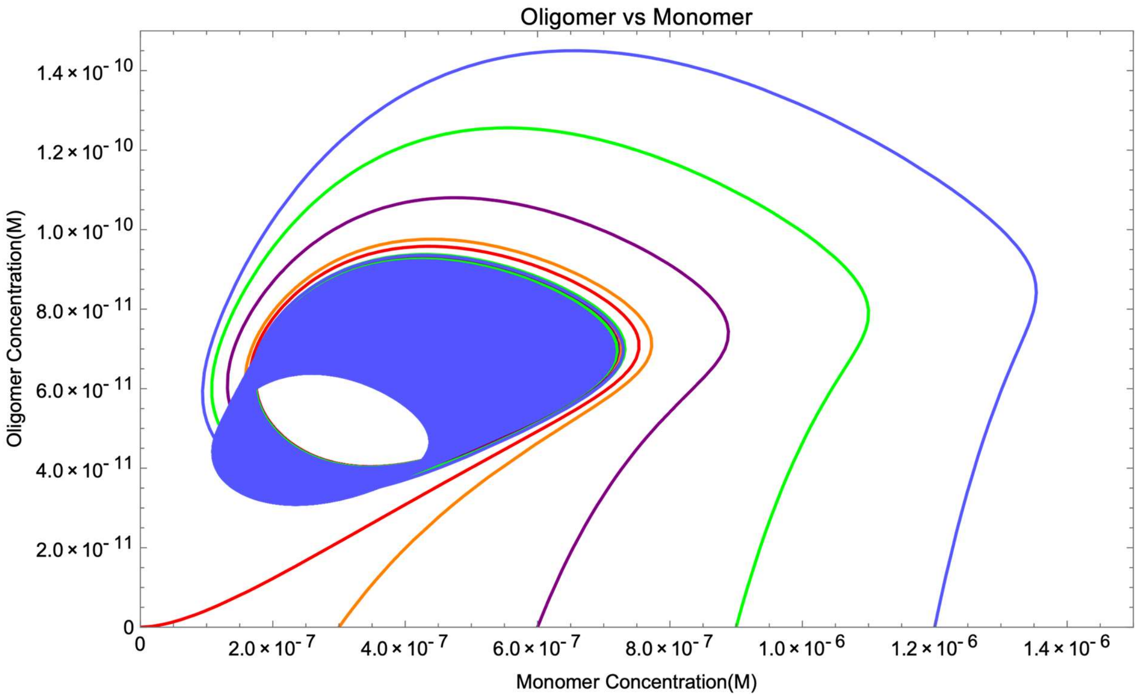

Supplementary Figure 7. The convergence of the oscillatory behaviors and the limit cycle of open coupled systems with different Aβ monomer concentrations (0 -> red; 0.3 μM -> orange, 0.6 μM-> purple, 0.9 μM-> light green, 1.2 μM -> blue).

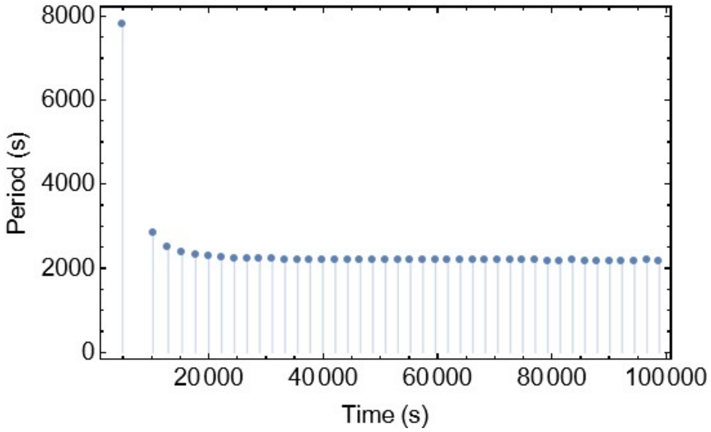

Supplementary Figure 8. The period of oscillation varies during the time course of simulations. In the case of steady oscillation, the period reaches an asymptotic value within the time course of the simulation.

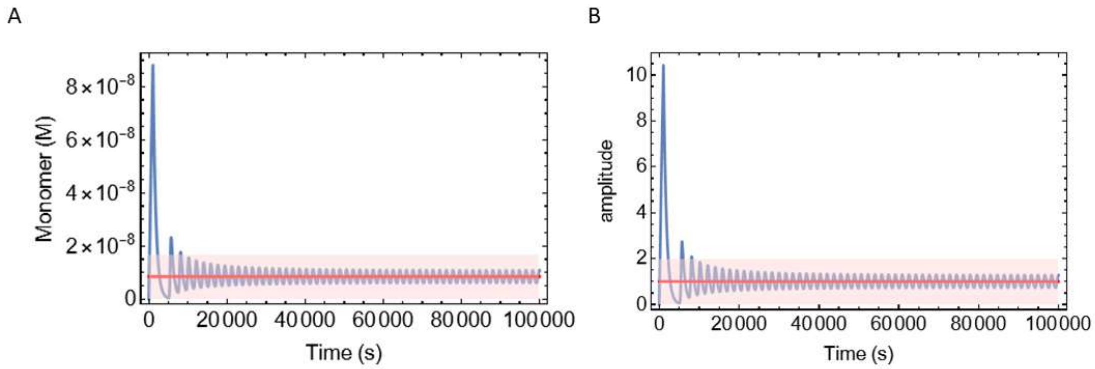

Supplementary Figure 9. The amplitude of oscillation is shown for a representative case of steady oscillation. (a) The baseline is calculated as the midpoint of the steady peaks and valleys of the oscillation after the initial period of stabilization, such that the baseline will assume the value of 1 (pink box). (b) The resultant normalized amplitudes can vary between the values of 0 and 2 (pink box).

Supplementary Table 1: Results of sensitivity analysis for the open uncoupled model. The numerical values represent the scaled sensitivity of the corresponding state variable (a_m, a_o, a_{fp}, a_f) *vs* different model parameters. *

	a_m	a_o	a_{fp}	a_f
k_+	0.955954	0.906956	10.3241	21.2092
k_-	-0.989641	-0.828797	-3.42233	-4.43007
γ_O	-0.0009986	-0.0150183	-0.0175357	-0.0183818
γ_f	1.49E-06	-0.0004099	-0.0001038	-0.0823782
j_1	-0.0032097	0.997391	0.991077	0.988581
j_{-1}	0.00316319	-0.987164	-0.973237	-0.96496
j_2	-0.0021791	0.00330252	0.998044	0.997033
j_3	-0.0021791	0.00330253	-0.0019536	0.997023
j_4	-1.60E-05	0.00503353	0.00168495	0.00086866

* a_m: monomeric Aβ ; a_o: oligomeric Aβ; a_{fp}: fibrillar Aβ (particle number concentration); a_f: fibrillar Aβ (mass concentration); For the meaning of parameters (first column), see the text.

Supplementary Table 2: Results of sensitivity analysis for the open coupled model. The numerical values represent the scaled sensitivity of the corresponding state variable (a_m, a_o, a_{fp}, a_f, *infl*) *vs* different model parameters. *

	a_m	a_o	a_{fp}	a_f	*infl*
k_{+0}	0.02883	0.01455	0.1782	0.40603	0.014586
$k_{+\infty}$	0	0	0	0	0
k_{-0}	-0.02333	-0.02279	-0.1261	228.818	-0.002
$k_{-\infty}$	-0.01292	-0.03962	-0.12496	219.167	-0.01971
γ_O	0.04333	-0.01194	0.11708	120.257	-0.00101
γ_f	-0.00048	-0.00039	0.00245	4.45198	1.06E-05
j_{10}	-0.48191	-0.00699	-1.35344	196.133	0.010828
$j_{1\infty}$	-0.74636	0.00579	-1.86037	120.944	0.016777
j_{-10}	0.4161	-0.02907	1.05527	216.875	-0.00936
$j_{-1\infty}$	0.67887	-0.03204	1.6867	184.672	-0.01526
j_{20}	-0.00093	-0.00076	0.39553	8.57599	2.04E-05
$j_{2\infty}$	-0.00092	-0.00076	0.61228	8.54691	2.04E-05
j_{30}	-0.00175	-0.00144	0.00759	16.2318	3.83E-05
$j_{3\infty}$	0.00037	0.00031	-0.00226	-3.48981	-8.29E-06
j_{40}	-0.00037	-0.00031	0.00174	3.45343	8.24E-06
$j_{4\infty}$	-0.00077	-0.00063	0.00353	7.11175	1.69E-05
δ_O	-1.21779	-0.99223	-4.22203	227.194	0.027417
δ_f	-0.00016	-0.00013	3.36E-05	0.42849	3.56E-06
k_{infl}	1.16939	0.95231	4.00556	235.928	-0.02631
$infl\_ref$	1.17927	0.96042	4.09029	144.291	0.973439
$steep$	-0.01402	-0.01143	-0.06082	27.1782	-0.00896

*a_m: monomeric Aβ ; a_o: oligomeric Aβ; a_{fp}: fibrillar Aβ (particle number concentration); a_f: fibrillar Aβ (mass concentration); *infl*: inflammation level; For the meaning of parameters (first column), see the text.
